# The effects of olive leaf extract on cardiovascular risk factors in the general adult population: a systematic review and meta-analysis of randomized controlled trials

**DOI:** 10.1186/s13098-022-00920-y

**Published:** 2022-10-21

**Authors:** Elham Razmpoosh, Shima Abdollahi, Mahdieh Mousavirad, Cain C. T. Clark, Sepideh Soltani

**Affiliations:** 1 Nutrition and Endocrine Research Center, Research Institute for Endocrine Sciences, Shahid Beheshti University of Medical Sciences, Tehran, Iran; 2 Department of Nutrition, School of Public Health, North Khorasan University of Medical Sciences, Bojnurd, Iran; 3 Yazd Cardiovascular Research Center, Noncommunicable Diseases Research Institute, Shahid Sadoughi University of Medical Sciences, Yazd, Iran; 4 Centre for Intelligent Healthcare, Coventry University, Coventry, CV1 5FB UK

**Keywords:** Olive leaf extract, Cardiovascular diseases, Blood pressure, Meta-analysis, Systematic review

## Abstract

**Background:**

The aim of this systematic review and meta-analysis was to determine the effect of olive leaf extract (OLE) supplementation on cardiovascular-related variables, including lipid, glycemic, inflammatory, liver and renal-related factors, as well as blood pressure.

**Methods:**

PubMed, ISI Web of Science, Scopus, and Cochrane library were searched, up to October 2021, for relevant controlled trials. Mean differences and standard deviations were pooled for all outcomes, using a random-effects model. The methodological quality, as well as quality of evidence were assessed using standard tools.

**Results:**

Twelve studies (n = 819 participants) were included in our analyses. Overall analyses showed that OLE supplementation significantly decreased triglyceride (TG) levels (WMD = − 9.51 mg/dl, 95% CI − 17.83, − 1.18; *P* = 0.025; *I*^2^ = 68.7%; *P*-heterogeneity = 0.004), and systolic blood pressure (SBP) (WMD = − 3.86 mmHg, 95% CI − 6.44, − 1.28 mmHg; *P* = 0.003; *I*^2^ = 19.9%; *P*-heterogeneity = 0.28). Subgroup analyses also revealed a significant improvement in SBP (− 4.81 mmHg) and diastolic blood pressure (− 2.45 mmHg), TG (− 14.42 mg/dl), total cholesterol (TC) (− 9.14 mg/dl), and low-density lipoprotein-C (LDL-C) (− 4.6 mg/dl) measurements, in patients with hypertension. Significant reductions were also observed in TC (− 6.69 mg/dl), TG (− 9.21 mg/dl), and SBP (− 7.05 mmHg) in normal-weight individuals. However, no meaningful changes were seen in glucose hemostasis, liver and kidney, or inflammatory markers.

**Conclusion:**

The present study revealed that supplementation with OLE yielded beneficial effects for blood pressure and lipid profile in adults, especially in patients with hypertension. As the quality of evidence for glucose hemostasis variables, liver, kidney, and inflammatory markers, were low-to-very low, higher quality RCTs may impact the overarching results.

*This study was registered at PROSPERO with the code CRD42022302395.*

**Supplementary Information:**

The online version contains supplementary material available at 10.1186/s13098-022-00920-y.

## Background

Cardiovascular disease (CVD) is one of the most prominent noncommunicable diseases (NCDs), accounting for the most NCD deaths in the world [1]. Modifiable unhealthy behaviors, such as sedentary lifestyle, smoking, and unhealthy food habits, are regarded as important contributors to the widespread prevalence of CVDs [2, 3], which occur concurrently in overweight/obesity, hypertension, dyslipidemia, hyperglycemia, and inflammation [3, 4].

Oxidative stress and chronic inflammation are among the biggest contributing factors in CVD pathogenesis and progression, and they have recently been introduced as the key targets for the prevention and treatment of CVDs [5]. Moreover, anomalies in glucose metabolism, such as elevated fasting blood glucose (FBG) and insulin resistance, as well as dyslipidemia and elevated blood pressure (BP), are demonstrably associated with a higher risk of CVD [2].

Meanwhile, interventional and epidemiological evidence supports the beneficial effects of antioxidants and antioxidant-rich diets on CVD risk factors [6–9]. Among different antioxidant-rich foods, olive oil, a typical component of the Mediterranean diet, is known as one of the most important health-protective agents, mainly due to its high content of polyphenols [10]. In addition to olive oil, leaves of the olive tree (known as *Olea europaea* L.) have been widely used in traditional remedies in Mediterranean countries [11]. The olive leaf extract (OLE) contains a high amount of phenolic antioxidant, named oleuropein, which is markedly higher than those found in olive fruit or olive oil [12, 13]. In recent years, the role of OLE in improvement of CVD-related variables has gained attention in clinical trial investigations in the general population, which mostly include its lipid-lowering [14, 15], anti-obesity [16], blood pressure-lowering [17], and anti-diabetic effects [18]. However, some other studies failed to show any significant improvements in body mass index (BMI) [19, 20], glucose hemostasis [17, 19], plasma lipids, [21], or cytokines [20]. Moreover, a previous meta-analysis of five human investigations reported that OLE supplementation did not have any significant effects on diastolic blood pressure (DBP), total cholesterol (TC), low-density lipoprotein cholesterol (LDL-C), and high-density lipoprotein cholesterol (HDL-C), and a slight improvement in systolic blood pressure (SBP) among patients with hypertension [22]. However, this study omitted two eligible studies [23, 24], and also did not examine the effect of OLE in the general population [22].

The exact mechanism of action of the health-related beneficial effects of OLE is not well understood; although some putative explanations have been proposed. Animal studies have showed that OLE exerts antidiabetic effects through increased peripheral glucose uptake, postprandial insulin secretion, and stimulation of glucagon-like peptide-1 (GLP-1) secretion [25, 26]. Furthermore, it has been shown that lipid peroxidation is inhibited through induced catalase activity following OLE supplementation in rats [27]. In addition, except for the oleuropein content of OLE, other constituents, such as hydroxytyrosol, are shown to have positive effects on glucose metabolism [28, 29].

Thus, we sought to investigate whether OLE could improve the major cardiovascular-related variables, including lipid profile, glucose hemostasis, blood pressure, as well as liver/kidney and inflammatory markers in the general adult population, by conducting a systematic review and meta-analysis of randomized clinical trials (RCTs).

## Methods

This systematic review and meta-analysis was prepared in accordance with the PRISMA (Preferred Reporting Items for Systematic Reviews and Meta-Analyses) guidelines [30] and was registered at PROSPERO with the code CRD42022302395.

### Data sources and search strategy

According to the PICOS tool for performing search strategies in systematic reviews and meta-analysis, endorsed by Cochrane Collaborations [31], the related components consisted of “adult populations” with any health conditions, OLE supplementation as the “intervention”, a concurrent placebo group as the comparator, and randomized controlled trial investigations as the “study design”. Accordingly, the related Medical Subject Headings (MeSH) and non-MeSH terms were used to search PubMed, ISI Web of Science, Scopus, and the Cochrane library, from inception to October, 2021. No restrictions were considered regarding the language, year of publication, the type of populations or the outcomes of measure. The reference lists of the included studies and related reviews were also checked to identify other potential missing studies. More details of the search strategy are provided in Additional file 1.

### Study selection

Two reviewers (MM and SS) independently reviewed the titles and abstracts of all records. Randomized-controlled trials were considered to be eligible if they: (1) had either a parallel or crossover design with at least two weeks of OLE supplementation; (2) included adult male or female participants aged 18 years and older, with any health condition; and (3) reported mean and standard deviation (SD) values of change (or provided sufficient data to calculate these variables) for at least one of the cardiovascular-related markers, including glucose indices, lipid profile, liver enzymes, kidney function, circulating inflammatory markers, and blood pressure, as the primary or secondary outcomes.

Studies were excluded if they: (1) used OLE or its active component (olea europaea) in combination with other interventions; (2) were conducted on children, adolescents, pregnant or lactating women; and/or (3) had an intervention duration of less than two weeks.

### Data extraction

Two authors (SS and MM) extracted the following information for each study: the last name of the first author, year of publication, study location, study design, doses (mg/d) and duration (week) of intervention, any other intervention given to groups, sample size in each group, sex (male or female), age (y), health status of participants, and means before and after the intervention or mean changes and the corresponding SDs during the follow-up period.

Regarding the articles that reported the outcomes of interest in the same set of population, we included the study that had the greatest sample size or the longest follow-up duration. Extracted data for each outcome were finally converted to a specified unit. Any disagreements were discussed with the corresponding author (SS).

### Study quality

Two reviewers (SS and MM) independently assessed the methodological quality of the eligible RCTs using the revised Cochrane risk-of-bias tool for randomized trials (RoB2), which consists of five main domains, including bias arising from the randomization process, bias due to deviations from the intended interventions, bias due to missing outcome data, bias in the outcome measures, and bias related to the selection of reported results. Final judgments and overall risk of bias were defined as “Low” or “High” risk of bias or expressed as “Some Concerns” [32].

### Certainty of evidence

The overall quality of evidence was evaluated, using the Grading of Recommendations Assessment, Development and Evaluation (GRADE) tool, independently by two reviewers (SS and MM). RCTs begin with a high quality of evidence, but the final quality may be downgraded by detecting the existence of study limitations, inconsistency, indirectness of evidence, and/or publication bias.

### Statistical analysis

All of the outcomes for this meta-analysis were reported as continuous data, for which we calculated the weighted mean difference (WMD), with their associated 95% CIs, as the absolute mean difference in change of the outcome of interest between OLE supplementation and the placebo arm. According to the Cochrane recommendations [33], for studies in which the mean and SD changes from baseline were not reported, mean changes were computed as the post-intervention mean minus the pre-intervention mean, and the SD values were yielded via computing the correlation coefficient from the study that reported baseline SD measures in each arm. The correlation coefficients were 0.61 for FBS [19, 23, 34, 35], 0.73 for TC [23, 36, 37], 0.60 for TG [19, 23, 36, 37], 0.71 for LDL-C, 0.79 for HDL-C [19, 23, 34, 37], 0.62 for interlukine-6 (IL-6), 0.60 for interlukine-8 (IL-8), 0.66 for tumor necrosis factor-α (TNF-α) [23, 35], 0.49 for SBP, and 0.52 for DBP [23, 36, 37], and 0.5 for other outcomes. The effect sizes were pooled using the inverse variance random-effects method, which took into account the heterogeneity among studies [38]. Statistical heterogeneity was examined using the Q (*P*-value < 0.1) and *I*^2^ statistics. The *I*^*2*^ represented moderate heterogeneity if ranging 30% to 50%, serious heterogeneity if ranging 50–75%, and very serious heterogeneity if ranging 75–100%[39]. The potential sources of heterogeneity between studies were explored by conducting a series of predefined subgroup analyses based on sex, study design, study duration, and health status of participants [defined as the presence of overweight/obesity, hypertension, and/or hyperlipidemia]. Subgroup analyses were conducted if four studies or more were included for each outcome.

The sensitivity analysis of every outcome was conducted by excluding one study or a group of studies at the same time to discern whether the selected study influenced the overall results. Publication bias was evaluated through Begg’s funnel plots [40] and Egger’s regression symmetry test [41], if more than 10 studies were included. All statistical analyses were performed using STATA software (version 16.0, Stata Corporation, College Station, TX, USA), and a *P* < 0.05 was, a priori, considered statistically significant.

## Results

### Literature flow

The primary search identified 723 articles, and after removing duplicates (n = 314), 344 records were excluded through screening of the titles and abstracts. We were unable to obtain the full-text of one article, despite contacting the corresponding author, and so, finally, 64 studies were reviewed in full-text. Twenty additional articles were excluded due to the irrelevant endpoints and design (Additional file 2. References 1–20). A further 32 articles were excluded for the following reasons: two studies were conducted on children (Additional file 2: References 21–22) and one among athletes (Additional file 2: Reference 23); ten articles applied multi-supplementation in the intervention group (Additional file 2: References 24–33); three studies used olives or olive extracts (Additional file 2: References 34–36), and four studies used olive pollen as the supplement (Additional file 2: References 37–40); one study was in-vitro research (Additional file 2: Reference 41), and two studies were food-industry investigations (Additional file 2: References 42–43). Seven studies did not consider any control group (Additional file 2: References 44–50); one study had insufficient data (Additional file 2: Reference 51), and another study had duplicate data from a previous publication (Additional file 2: Reference 52).

**Fig. 1:**
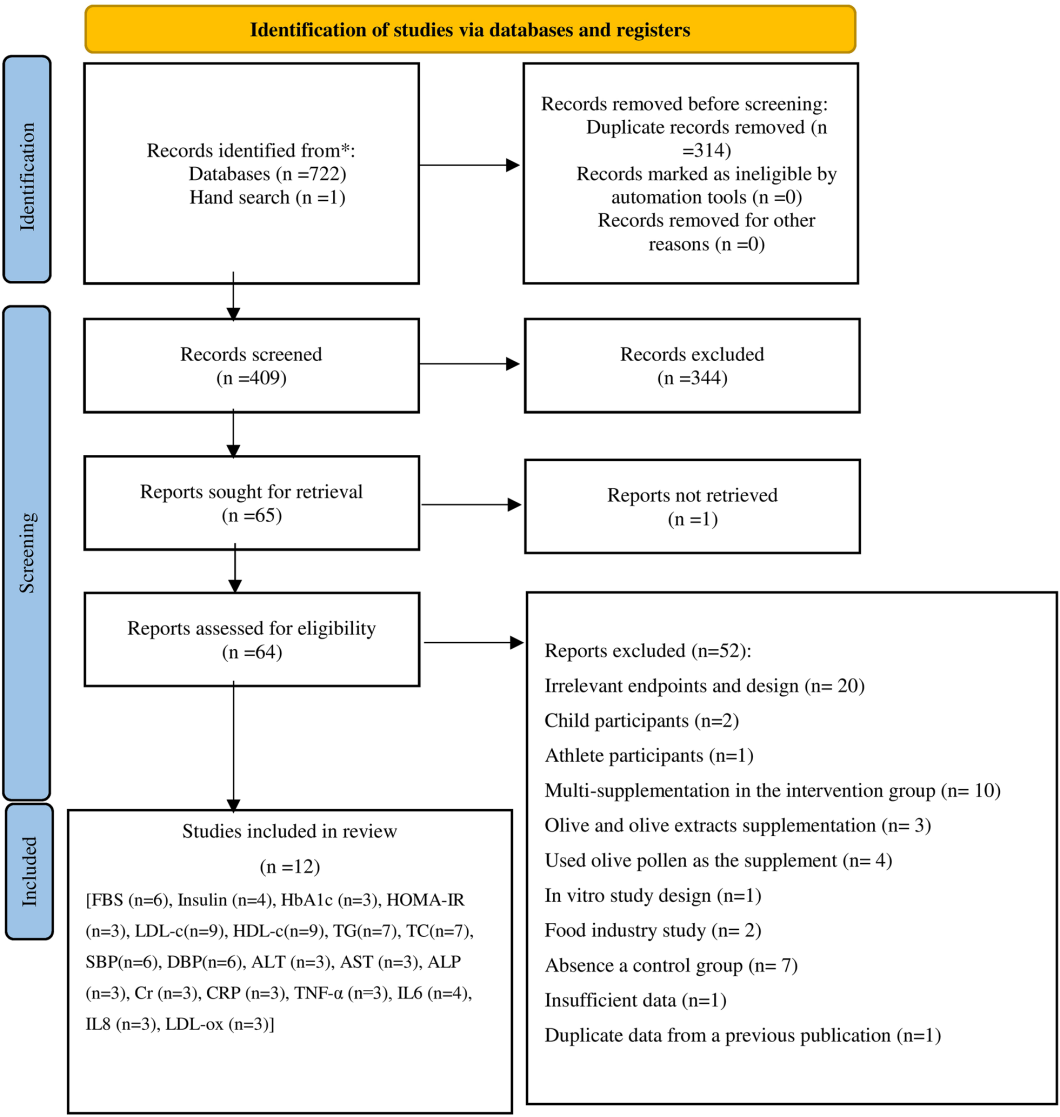
Flow diagram for the study selection process

Finally, twelve eligible studies were included in the systematic review and meta-analysis [19–21, 23, 24, 34–37, 42–44]. The study selection process is presented in Fig. 1.

### Characteristics of the included studies

The characteristics of the included studies are outlined in Table 1. All of the included studies were in English, except for three, which were in Japanese [34] and Farsi [24, 37]. Studies were conducted in Iran [24, 35, 37], the Netherlands [42, 43], Australia and New Zealand [20, 23], Japan [19, 34], Switzerland [21], Indonesia [36], and Israel [44]. Except for two studies that used the liquids [23] or beverages [34] of OLE, the rest of the studies used either tablets or capsules of OLE. All but two studies applied a parallel design [20, 23]. The duration and dose of OLE supplementation varied from 6 to 48 weeks, and 500 mg to 5 g per day, respectively. Two studies included males [20, 23], one study included females [42], and the rest of the studies enrolled both males and females. Studies were conducted among patients with type-2 diabetes mellitus [19, 44], hypertension [21, 23, 24, 35–37], dyslipidemia [34, 43], obesity [20], and osteopenia [42]. Eight studies considered placebo controls in their investigations [20, 21, 23, 24, 35, 37, 43, 44], and one study used a low concentration of green tea as the comparator [34]. One study examined the effect of OLE supplementation in combination with calcium, in which the calcium supplements were given to both the intervention and the control groups [42]. One investigation used captopril in the control group, which was excluded in the analysis of blood pressure [36]. None of the included studies assessed the pure bioactive compounds of OLE, and none reported any related adverse effects.

**Table 1:** Characteristics of eligible randomized controlled trials that investigated the effect of OLE intervention on cardiovascular-risk factors in adults

Author, year	Country	Participants (I, C)/sex	Design/duration (weeks)	Mean age	Health status					Intervention protocol/control protocol	Outcome
					Obesity	HTN	Dyslipidemia	T2DM	Other		
Araki, 2019 [19]	Japan	(28, 29)/B	P/12	52	Y	N	N	Y	–	1000 ml OLT (32.4 mg/100 g Oleuropein, 1.2 mg/100 g Hydroxytyrosol)/0.5 g LOLT ((2.4 mg/100 g Oleuropein, 0.3 mg/100 g HT)	FBS, HbA1c, HOMA-IR, LDL-C, HDL-C,
Araki, 2018 [34]	Japan	(20, 19)/B	P/12	52	N	N	Y	N	–	1000 ml OLT (425 mg/L Oleuropein,6.4 mg/L Hydroxytyrosol)/Green tea (0 mg/L Oleuropein, and 4.9 mg/L Hydroxytyrosol)	FBS, HbA1c, LDL-C, HDL-C, TG
de Bock, 2013 [20]	New Zealand	(45, 45)/M	C/12	46.5	Y	N	N	N	–	4 Cap (51.1 mg Oleuropein, 9.6 mg HT)/ Pl	LDL-C, HDL-C, TG, TC, SBP, DBP, hs-CRP, IL-6, IL-8, TNF-α, LDL-ox,
Filip, 2015 [42]	Netherlands	(32, 32)/F	P/48	59.7	N	N	N	N	Postmenopausal women with osteopenia	250 mg Cap (40% ≤ Oleuropein) + 400 mg Ca/400 mg Ca	LDL-C, HDL-C, TG, TC, hs-CRP, IL-6, ALP, Cr
Javadi, 2019 [35]	Iran	(30, 30)/B	P/12	54	N	Y	N	N	–	500 mg Tab (16% Oleuropein, 0.62 mg luteolin)/Pl	FBS, Ins, HOMA-IR, IL-6, IL-8, TNF-α, AST, ALT, ALP, Cr
Lockyer, 2017 [17]	Australia	(31, 30)/M	C/6	45.3	Y	Y	N	N	–	20 ml/day OLE liquid (equaled to 136.2 mg/day oleuropein and 6.4 mg/day Hydroxytyrosol)/Pl	FBS, Ins, HOMA-IR, LDL-C, HDL-C, TG, TC, SBP, DBP, hs-CRP, IL-6, IL-8, TNF-α, LDL-ox
Perrinjaquet-Moccetti, 2008 [21]	Switzerland	(10, 10)/B	P/8	36	N	Y	N	N	–	500 mg Tab OLE (e 18–26%) oleuropein, 30–40% polyphenols and luteolin-7-glucoside)/Pl	LDL-C, HDL-C, TC, SBP, DBP
Saberi, 2018 [24]	Iran	(32, 32)/B	P/NM	54	N	Y	N	N	–	1000 mg Cap OLE/Pl	SBP, DBP,
Stevense, 2021 [43]	Netherlands	(39, 38)/B	P/8	56.5	Y	N	Y	N	–	500 mg OLE (16.7%, and 83.5 mg oleuropein)/Pl	FBS, Insulin, LDL-C, HDL-C, TG, TC, SBP, DBP, LDL-ox, AST, ALT, ALP
Susalit, 2011 [36]	Indonesia	(72, 76)/B	P/8	50	N	Y	N	N	–	500 mg Caplet (19.9%) oleuropein)/12.5 mg captopril	FBS, Insulin, LDL-C, HDL-C, TG, TC, AST, ALT, Cr, SBP, DBP
Wainstein, 2012 [44]	Israel	(41, 38)/	P/14	≥ 55	N	N	N	Y	–	500 mg Tab OLE /Pl	HbA1c
Yaghoobzadeh, 2020 [37]	Iran	(30, 30)/B	P/12	54.5	N	Y	N	N	–	500 mg Tab OLE (16% and 0.6 luteolin)/Pl	LDL-C, HDL-C, TG, TC, SBP, DBP

P, parallel design; C, crossover design; HTN, hypertension; T2DM, type-2 diabetes mellitus; OLT, olive leaf extract; OLT, olive leaf tea; LOLT, low concentration of olive leaf tea; FBS, fasting blood glucose; HbA1c, glycated hemoglobin; HOMA-IR, homeostatic model assessment for insulin resistance; LDL-C, low-density lipoprotein cholesterol; HDL-C, high-density lipoprotein cholesterol; TG, triglycerides; TC, total cholesterol; SBP, systolic blood pressure; DBP, diastolic blood pressure; hs-CRP, high-sensitivity C-reactive protein; IL-6, interleukin-6; IL-8, interleukin-8; TNF-α, tumor necrosis factor alpha; LDL-ox, oxidized low-density lipoprotein; AST, aspartate transaminase; ALT, alanine transaminase; ALP, alkaline phosphatase; Cr, creatinine; Cap, capsule; Tab, tablet; Ca, calcium, PI, per intervention

### Risk of bias and quality of evidence

According to the overall quality assessment based on the Cochrane Collaboration Risk of Bias tool (Additional file 3), six studies were classified as “low” risk of bias (i.e., low risk of bias for all domains) [20, 23, 36, 37, 42, 43], three studies were classified as “some concerns” [24, 35, 44], and the three remaining studies were classified as “high” risk of bias [19, 21, 34]. To explain the details of the observed biases, three studies did not clearly explain the randomization and allocation concealment processes [19, 21, 34]. The blinding process was not considered in one study [21] and was not clearly explained in two investigations [19, 34]. One study had a “high” risk of bias [21] and three studies had “some concerns” risk of bias [19, 34, 35] due to the measurements of the outcomes where outcome assessors were not blinded to the study. One study did not clearly report the number of participants with missing outcome data [21] and three studies [21, 24, 44] were also shown to have insufficient data regarding the pre-specified analysis plan (bias due to the selection of the reported results).

According to the evaluation of the quality of evidence based on the GRADE system, the quality of evidence was found to be very low for the effect of OLE supplementation on HbA1c a, high sensitive C-reactive protein (hs-CRP), TNF-α, IL-6, and IL-8. A low quality of evidence was also observed for insulin, AST, ALT, ALP, and creatinine, as well as LDL-ox. A moderate quality of evidence was observed for that of OLE supplementation on FBS, LDL-C, HDL-C, TC, TG, and blood pressure (SBP and DBP) levels (Additional file 3).

### Meta-analysis

#### Lipid profile

##### TC

**Fig. 2:**
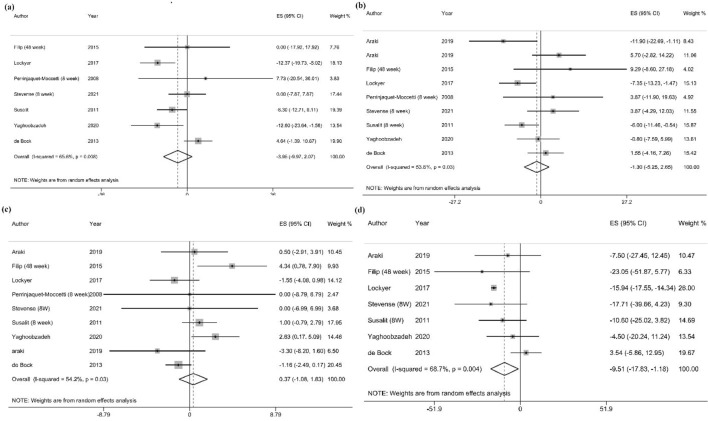
Forest plot of randomized controlled trials (RCTs) illustrating weighted mean differences in **a** total cholesterol, **b** LDL-c, **c** HDL-c, and **d** triglyceride between OLE supplementation and control groups for all eligible studies. Analysis was conducted using a random effects model

Meta-analysis of seven studies [20, 21, 23, 36, 37, 42, 43], including 520 participants, showed that OLE supplementation had no significant effect on the levels of TC, and the heterogeneity between studies was found to be moderate (WMD = − 3.95 mg/dl, 95% CI − 9.97, 2.07; *P* = 0.2; *I*^2^ = 65.6%; *P*-heterogeneity = 0.008) (Fig. 2a). Subgroup analyses showed a significant reducing effect of OLE supplementation on TC levels in normal-weight participants (4 studies; WMD = − 6.69 mg/dl, 95% CI − 11.90, − 1.49; *P* = 0.01; *I*^2^ = 0.0%; *P*-heterogeneity = 0.45), and in patients with hypertension (4 studies; WMD = − 9.14 mg/dl, 95% CI − 13.80, − 4.47; *P* < 0.001; *I*^2^ = 7.5%; *P*-heterogeneity = 0.36) (Additional file 4).

##### LDL-C

Nine RCTs [19–21, 23, 34, 36, 37, 42, 43], including 616 participants, evaluated the effect of OLE on LDL-C levels. Results indicated that OLE supplementation yielded in an insignificant reduction in LDL-C measures and the between-study heterogeneity was reported to be medium (WMD = − 1.30 mg/dl, 95% CI − 5.25, 2.65; *P* = 0.52; *I*^2^ = 53.6%; *P*-heterogeneity = 0.03) (Fig. 2b). Subgroup analyses showed that LDL-C concentration decreased significantly in patients with hypertension (4 studies; WMD = − 4.60 mg/dl, 95% CI − 8.26, − 0.94; *P* = 0.014; *I*^2^ = 11.7%; *P*-heterogeneity = 0.33). Other potential sources of heterogeneity are reported in Additional file 4.

##### HDL-C

Among the included studies, nine investigations [19–21, 23, 34, 36, 37, 42, 43] with 616 participants assessed the effect of OLE on HDL-C concentrations and reported no significant related changes (WMD = 0.38 mg/dl, 95% CI − 1.08, 1.83; *P* = 0.61; *I*^2^ = 54.2%; *P*-heterogeneity = 0.03) (Fig. 2c). According to the subgroup analyses, the levels of HDL-C decreased significantly in male participants following OLE supplementation (2 studies; WMD = − 1.24 mg/dl, 95% CI − 2.42, − 0.07; *P* = 0.04; *I*^2^ = 0.0%; *P*-heterogeneity = 0.79). Additional file 4 shows further potential sources of heterogeneity.

##### TG

According to the pooled analysis of seven studies (539 participants) [20, 23, 34, 36, 37, 42, 43], OLE supplementation resulted in a significant decrease in TG levels with a moderate between-study heterogeneity (WMD = -9.51 mg/dl, 95% CI − 17.83, − 1.18; *P* = 0.025; *I*^2^ = 68.7%; *P*-heterogeneity = 0.004) (Fig. 2d). Subgroup analyses showed that OLE supplementation significantly decreased TG levels in participants with a normal body weight (4 studies, WMD = − 9.21 mg/dl, 95% CI − 18.14, − 0.29; *P* = 0.04; *I*^2^ = 0.0%; *P*-heterogeneity = 0.73), and participants with hypertension (3 studies, WMD = − 14.32 mg/dl, 95% CI − 19.36, − 9.28; *P* < 0.001; *I*^2^ = 20.2%; *P*-heterogeneity = 0.28) (Additional file 4).

#### Glucose homeostasis

**Fig. 3:**
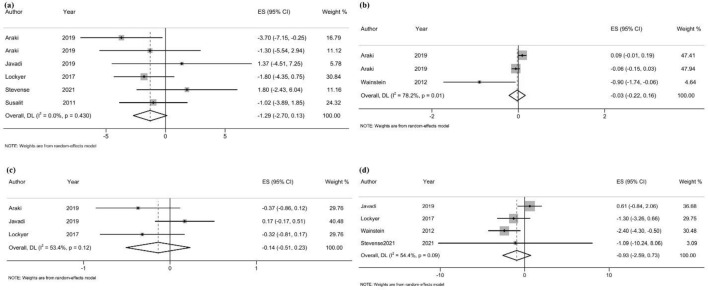
Forest plot of randomized controlled trials (RCTs) illustrating weighted mean differences in **a** FBS, **b** HbA1c, **c** HOMA-IR, and **d** insulin levels between OLE supplementation and control groups for all eligible studies. Analysis was conducted using a random effects model

In total, six RCTs [19, 23, 34–36, 43], with a total of 442 participants, examined the effect of OLE supplementation on FBS levels, and according to the pooled analysis, there was an insignificant reduction in blood FBS concentration (WMD = − 1.29 mg/dl, 95% CI − 2.70, 0.13; *P* = 0.07; *I*^2^ = 0.0%; *P*-heterogeneity = 0.43) (Fig. 3a). Based on the subgroup analyses, FBS levels decreased significantly after OLE supplementation in participants without dyslipidemia (4 studies; WMD = -1.73 mg/dl, 95% CI − 3.33, − 0.13; *P* = 0.03; *I*^2^ = 0.0%; *P*-heterogeneity = 0.47) (Additional file 4).

Moreover, pooling effect sizes from three RCTs (175 participants) [23, 35, 43, 44] showed no significant effect of OLE supplementation on HbA1c (Fig. 3b) (WMD = − 0.03%, 95% CI − 0.22, 0.16; *P* = 0.77; *I*^2^ = 78.2%; *P*-heterogeneity = 0.01), HOMA-IR (Fig. 3c) (3 studies, n = 221; WMD = − 0.14, 95% CI − 0.51, 0.0; *P* = 0.47; *I*^2^ = 53.4%; *P*-heterogeneity = 0.12), and blood insulin levels (Fig. 3d) (4 studies, n = 320 participants; WMD = − 0.93 μU/L, 95% CI − 2.59, 0.73; *P* = 0.27; *I*^2^ = 54.4%; *P*-heterogeneity = 0.09).

#### Blood pressure

##### SBP

**Fig. 4:**
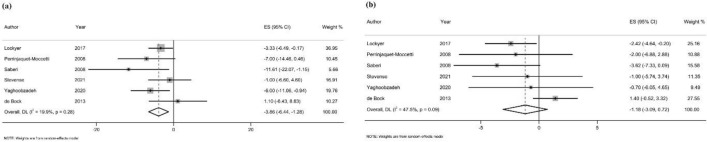
Forest plot of randomized controlled trials (RCTs) illustrating weighted mean differences in **a** SBP, and **b** DBP between OLE supplementation and control groups for all eligible studies. Analysis was conducted using a random effects model

Six studies [20, 21, 23, 24, 37, 43] examined the effect of OLE supplementation on the SBP measure (n = 372 participants). One study was excluded from the final analyses, since the control group received an anti-hypertension treatment [36]. The pooled effect sizes showed a significant reduction in SBP after OLE supplementation (WMD = − 3.86 mmHg, 95% CI − 6.44, − 1.28; P = 0.003; *I*^*2*^ = 19.9%; *P*-heterogeneity = 0.28) (Fig. 4a). Subgroup analyses also revealed that SBP was reduced significantly following OLE supplementation in participants with a normal lipid profile (4 studies; WMD = − 4.47 mmHg, 95% CI − 7.39, − 1.56; P = 0.003; *I*^*2*^ = 22.2%; *P*-heterogeneity = 0.27), individuals with normal body weight (3 studies; WMD = − 7.05 mmHg, 95% CI − 10.94, − 3.16; *P* < 0.001; *I*^*2*^ = 0.0%; *P*-heterogeneity = 0.64), and patients with hypertension (4 studies; WMD = − 4.81 mmHg, 95% CI − 7.27, − 2.35; *P* < 0.001; *I*^*2*^ = 0.3%; *P*-heterogeneity = 0.39) (Additional file 4).

##### DBP

Pooled analyses of six studies [20, 21, 23, 24, 37, 43] (n = 372 participants) showed that OLE supplementation did not have any significant effect on DBP (WMD = − 1.18 mmHg, 95% CI − 3.09, 0.72; *P* = 0.22; *I*^2^ = 47.5%; *P*-heterogeneity = 0.09) (Fig. 4b). However, based on the subgroup analyses, a significant decrease in DBP was observed in participants with hypertension (4 studies; WMD = − 2.45 mmHg, 95% CI − 4.13, − 0.76; *P* = 0.004; *I*^2^ = 0.0%; *P*-heterogeneity = 0.84). Other possible sources of between-study heterogeneity are shown in Additional file 4.

#### Liver and kidney variables

##### Liver enzymes

According to the pooled analyses, no significant changes with no evidence of heterogeneity were observed in ALP (Additional file 5) [3 studies [35, 42, 43], n = 184; WMD = -0.41 μU/L, 95% CI − 6.06, 5.23; *P* = 0.89; *I*^2^ = 0.0%; *P*-heterogeneity = 0.47], AST (Additional file 5) [3 studies [35, 36, 43], n = 315; WMD = 0.22 μU/L, 95% CI − 1.63, 2.06; *P* = 0.47; *I*^2^ = 0.0%; *P*-heterogeneity = 0.68], and ALT levels (Additional file 5) [3 studies [35, 36, 43], n = 315; WMD = 0.33 μU/L, 95% CI − 0.96, 1.62; *P* = 0.62; *I*^2^ = 0.0%; *P*-heterogeneity = 0.763], after OLE supplementation. We were unable to perform subgroup analyses due to the limited number of studies.

##### Creatinine

Pooling data from three eligible studies [20, 23, 42] showed no significant related change in creatinine levels and no evidence of heterogeneity between studies (n = 285; WMD = − 0.03 mg/dl, 95% CI − 0.09, 0.03; *P* = 0.35; *I*^2^ = 0.0%; *P*-heterogeneity = 0.45) (Additional file 5).

#### Inflammatory markers

Compared to the control group, OLE supplementation had no significant effect on any of the inflammatory markers, including hs-CRP (Additional file 5) (3 studies, n = 238; WMD = 0.24 mg/dl, 95% CI − 0.20, 0.68; *P* = 0.28; *I*^2^ = 63.9%; *P*-heterogeneity = 0.06), IL-6 (Additional file 5) (4 studies, n = 220; WMD = 0.02 pg/ml, 95% CI − 0.28, 0.33; *P* = 0.88; *I*^2^ = 58.1%; *P*-heterogeneity = 0.07), IL-8 (Additional file 5) (3 studies, n = 188; WMD = -0.36 pg/ml, 95% CI − 0.99, 0.27; *P* = 0.26; *I*^2^ = 82.7%; *P*-heterogeneity = 0.003), TNF-α (Additional file 5) (3 studies, n = 186; WMD = − 0.31 pg/ml, 95% CI − 0.93, 0.32; *P* = 0.34; *I*^2^ = 75.1%; *P*-heterogeneity = 0.02), and LDL-ox (Additional file 5) (3 studies, n = 267; WMD = − 2.01 pg/ml, 95% CI − 5.78, 1.77; *P* = 0.30; *I*^2^ = 0.0%; *P*-heterogeneity = 0.47). There was some evidence of heterogeneity; however, the sources of heterogeneity remained unknown due to the low number of studies.

### Sensitivity analysis and publication bias

A leave-one-out sensitivity analysis was performed to identify the influential studies. However, effect estimates remained stable for all outcomes. We also conducted the sensitivity analysis excluding studies with high risk of bias (studies with less quality), and the results remained unchanged (Additional file 6).

Publication bias was not assessed, since the number of included studies in each outcome was less than 10.

## Discussion

This systematic review and meta-analysis of twelve RCTs indicated that OLE supplementation significantly decreased TG and SBP levels. The main results of subgroup analyses revealed that OLE may improve lipid profile and blood pressure more effectively in participants with hypertension and normal body weight.

A comprehensive pooling data demonstrated that BP-lowering treatments are associated with a lower risk for death and CVD events, especially when baseline SBP is 140 mmHg or higher [45]. Another large-scale meta-analysis found that a reduction of 5-mmHg in SBP was associated with a decreased risk of major CVD events by 10%, irrespective of previous diagnoses of CVD [46]. In accordance with a previous meta-analysis [22], we observed a 4.81 mmHg reduction in SBP following OLE supplementation in patients with hypertension, suggesting that OLE supplementation may be useful as an adjunct therapy in these patients. Based on the subgroup analyses, the conclusion that patients with hypertension would benefit more from OLE may also suggest that there would be a common mechanism through which OLE exert its beneficial effects on blood pressure as well as the other common risk factors for CVD.

The present study indicated that OLE supplementation only has short-term positive effects on blood pressure and lipid profiles, which may be attributed to the active constituents in OLE [47]. In particular, oleuropein and oleacein, are the major components of OLE, and are reported to possess acute anti-hypertensive activity through inhibition of angiotensin-converting enzyme (ACE) [48]. Furthermore, previous research showed that oleuropein has a short-term vasodilatory effect [49], as well as a direct calcium antagonistic action [50]. On the other hand, oleuropein has been identified as a ligand of the peroxisome proliferator-activated receptor-alpha (PPAR-α) [51]. Studies have found that PPAR-α agonists can effectively modulate lipid profile, especially TG, and these agonists are currently being used as important targets for the treatment of insulin resistance and dyslipidemia [52]. Furthermore, PPAR-α activation favorably downregulates the expression of proinflammatory genes and affects serum lipid levels. This may also justify our results regarding an insignificant reduction in TG levels when the analysis was restricted to obese participants; indeed, as previously stated, the beneficial effects of OLE on lipid profile have been observed in the short term, and because the adipose tissue increases inflammation, longer durations and higher doses of OLE supplementation may be needed to detect a significant difference. On the other hand, obese participants predominantly need lifestyle modifications and specific medications, rather than taking adjunct therapies such as OLE supplements [21]. Moreover, it has been shown that oleuropein decreases the activity of hydroxymethylglutaryl-CoA reductase, leading to a reduction in cholesterol synthesis in hepatocytes of rat [15]. However, surprisingly, we found that HDL-C levels decreased in men after OLE supplementation. It is of note that only two studies were included in this subgroup, which enrolled obese participants, and thus, a potential adverse effect in obese men should be monitored.

Our analyses failed to detect any significant changes in glucose hemostasis variables, as well as the inflammatory and liver/kidney markers. The most likely reason might be related to the limited number of included studies. Moreover, the baseline measures of these variables were within normal ranges across the included studies, except for two investigations which were conducted among patients with diabetes and pre-diabetes [19, 44]. Besides, the variable baseline characteristics of participants could be a further contributory factor to the null results, including physical activity, alcohol consumption, smoking, dietary fat intake and etc., which were not reported in all of the included studies [53]. The quality of evidence was shown to be low and very low for these outcomes, and more high-quality studies may change the results in the future.

In the present study, we used a standard methodology to perform a systematic review and meta-analysis to answer the question of whether OLE supplementation has an effect on cardiometabolic factors. To do this, we designed a comprehensive search strategy without considering the outcomes of measures to ensure that we found all the relevant studies. Although a previous meta-analysis tried to answer this question [22], serious limitations were present, including missing two eligible RCTs [23, 24] and splitting analyses based on the dose of OLE supplementation. In our study, various subgroups and sensitivity analyses were performed to determine the sources of heterogeneity, and we examined the methodological quality and the quality of evidence using standard tools.

Notwithstanding our methodological rigor, the majority of the assessed outcomes had low quality of evidence and nearly half of the included studies had either a moderate or high risk of bias. This might also be the main reason for the observed heterogeneous findings in the subgroup analyses. Moreover, evidence showed that the GRADE evaluation relies on metrics for judging heterogeneity and incoherence and may lack quantitative evaluation criteria [54]. In addition, we were unable to perform subgroup analyses for some of our interested outcomes including liver, kidney, and inflammatory markers, as well as insulin levels, due to the limited number of studies. This limitation also prevented us from examining the publication bias. Moreover, some of the included studies did not report the pure concentration of oleuropein, a bioactive component of OLE, which further limited us to perform subgroup analyses based on the concentration of oleuropein and determine which precise dose had the most favorable effects on the common risk factors associated with CVD. Additionally, although we considered some confounding factors such as duration of the intervention, baseline BMI, and the health status of participants in the subgroup analyses, many confounding factors, including dietary intakes, physical activity, alcohol consumption, and smoking were not taken into account in the final analyses.

## Conclusion

The present systematic review and meta-analysis revealed that supplementation with OLE had a significant beneficial effect on TG and SBP in adults. Furthermore, we found that supplementation with OLE had more profitable effects on the improvement of TG, SBP, DBP, TC, and LDL-C measures among participants with hypertension and individuals with normal body weight. However, no meaningful changes were found in glucose hemostasis, liver and kidney variables, or inflammatory markers. Stronger RCT investigations, assessing different doses and durations of OLE, are required to better elucidate the effects of OLE supplementation.

## Supplementary Information

**Additional file 1:** Search strategy and table of excluded studies.

**Additional file 2:** References of excluded studies.

**Additional file 3:** Study quality and risk of bias assessment using Cochrane collaboration tool.

**Additional file 4:** Meta-analysis showing the effect of OLE supplementation on total cholesterol based on several subgroups.

**Additional file 5:** Forest plot of randomized controlled trials illustrating weighted mean differences in liver enzymes and creatinine.

**Additional file 6:** Meta-analysis showing the effect of OLE supplementation on cardiovascular risk factors including studies with good quality.

## Data Availability

The datasets generated or analyzed during the current study are available from the corresponding author on reasonable request.
