# Supplementary material for: The effects of olive leaf extract on cardiovascular risk factors in the general adult population: a systematic review and meta-analysis of randomized controlled trials
Source: Diabetol Metab Syndr. 2022 Oct 21;14:151. doi: 10.1186/s13098-022-00920-y (PMC9585795; doi:10.1186/s13098-022-00920-y)
Supplement: Supplementary file 1 — Additional file 1: Search strategy and table of excluded studies. [file 13098_2022_920_MOESM1_ESM.docx]

**Additional file 1: Search strategy and table of excluded studies**

*Search strategy*

PubMed, Scopus, ISI web of Science and the Cochrane library were searched, using the combination of the following components:

Component 1: Olive leaf Extract (as the intervention)

"olive leaf extract" [Online ResourceConcept] OR “Olive leaf extract”[Title/Abstract] OR “Olive leaves extract”[Title/Abstract] OR “Olive leaf”[Title/Abstract] OR “olea europaea”[Title/Abstract]

Component 2: Randomized controlled trial (as the study design)

Randomized [Title/Abstract] OR random[Title/Abstract] OR "Random allocation"[Title/Abstract] OR "Random assignment"[Title/Abstract] OR Intervention[Title/Abstract] OR "Clinical trial"[Title/Abstract] OR "Randomized controlled trial"[Title/Abstract] OR "Randomized controlled trials"[Title/Abstract] OR trial[tiab] OR Placebo [Title/Abstract] OR "Double-blind"[Title/Abstract] OR "Single-blind" [Title/Abstract] OR Clinical trial [Mesh] OR "Random Allocation" [Mesh] OR Randomised [Title/Abstract] OR "Randomised clinical trials"[Title/Abstract] OR "Randomised clinical trial"[Title/Abstract]

*Table of excluded studies*

Studies were excluded due to the:

| - Irrelevant endpoints and design (n= 20) (1-20) |
| --- |
| - Child participants (n=2) (21, 22) |
| - Athlete participants (n=1) (23) |
| - Multi-supplementation in the intervention group (n= 10) (24-33) |
| - Olive and olive extracts supplementation (n= 3) (34-36) |
| - Used olive pollen as the supplement (n= 4) (37-40) |
| - In vitro study design (n=1) (41) |
| - Food industry study (n= 2) (42, 43) |
| - Absence a control group (n= 7) (44-50) |
| - Insufficient data (n=1) (51) |
| - Duplicate data from a previous publication (n=1) (52) |
