# Supplementary material for: The effects of olive leaf extract on cardiovascular risk factors in the general adult population: a systematic review and meta-analysis of randomized controlled trials
Source: Diabetol Metab Syndr. 2022 Oct 21;14:151. doi: 10.1186/s13098-022-00920-y (PMC9585795; doi:10.1186/s13098-022-00920-y)
Supplement: Supplementary file 2 — Additional file 2: References of excluded studies. [file 13098_2022_920_MOESM2_ESM.docx]

**Additional file 2: References of excluded studies**

1. Kerimi A, Nyambe-Silavwe H, Pyner A, Oladele E, Gauer JS, Stevens Y, et al. Nutritional implications of olives and sugar: attenuation of post-prandial glucose spikes in healthy volunteers by inhibition of sucrose hydrolysis and glucose transport by oleuropein. European journal of nutrition. 2019;58(3):1315-30.

2. Lockyer S, Corona G, Yaqoob P, Spencer JPE, Rowland I. Secoiridoids delivered as olive leaf extract induce acute improvements in human vascular function and reduction of an inflammatory cytokine: a randomised, double-blind, placebo-controlled, cross-over trial. British Journal of Nutrition. 2015;114(1):75-83.

3. Perugini P, Vettor M, Rona C, Troisi L, Villanova L, Genta I, et al. Efficacy of oleuropein against UVB irradiation: Preliminary evaluation. Int J Cosmet Sci. 2008;30(2):113-20.

4. Takeda R, Koike T, Taniguchi I, Tanaka K. Double-blind placebo-controlled trial of hydroxytyrosol of Olea europaea on pain in gonarthrosis. Phytomedicine : international journal of phytotherapy and phytopharmacology. 2013;20(10):861-4.

5. Toulabi T, Delfan B, Rashidipour M, Yarahmadi S, Ravanshad F, Javanbakht A, et al. The efficacy of olive leaf extract on healing herpes simplex virus labialis: A randomized double-blind study. The journal of nutrition, health & aging. 2021.

6. Williamson G, Ferdousi F, Araki R, Hashimoto K, Isoda H. Olive leaf tea may have hematological health benefit over green tea. Nutrients. 2019;38(6):2952-5.

7. Boss A, Kao CH, Murray PM, Marlow G, Barnett MP, Ferguson LR. Human Intervention Study to Assess the Effects of Supplementation with Olive Leaf Extract on Peripheral Blood Mononuclear Cell Gene Expression. International journal of molecular sciences. 2016;17(12).

8. Kosaraju SL, D'Ath L, Lawrence A. Preparation and characterisation of chitosan microspheres for antioxidant delivery. Carbohydrate Polymers. 2006;64(2):163-7.

9. Rosalino LM, Loureiro F, Macdonald DW, Santos-Reis M. Food digestibility of an Eurasian badger Meles meles with special reference to the Mediterranean region. Acta Theriologica. 2003;48(2):283-8.

10. Castejón ML, Montoya T, Alarcón-de-la-lastra C, Sánchez-hidalgo M. Potential protective role exerted by secoiridoids from olea europaea l. In cancer, cardiovascular, neurodegenerative, aging-related, and immunoinflammatory diseases. Antioxidants. 2020;9(2).

11. D Del Rio 1 LGC, M E J Lean, A Crozier. Polyphenols and health: what compounds are involved? 2010.

12. Gomez-Cansino R, Guzman-Gutierrez SL, Campos-Lara MG, Espitia-Pinzon CI, Reyes-Chilpa R. Natural Compounds from Mexican Medicinal Plants as Potential Drug Leads for Anti-Tuberculosis Drugs. Anais Da Academia Brasileira De Ciencias. 2017;89(1):31-43.

13. Sindi HA. Evidence that supports the antidiabetic, antihypertensive, and antihyperlipidemic effects of olive (Olea europaea L.) leaves extract and its active constituents (oleuropein) in human. Journal of Biochemical Technology. 2020;11(2):41-5.

14. Vaughn AR, Clark AK, Sivamani RK, Shi VY. Natural Oils for Skin-Barrier Repair: Ancient Compounds Now Backed by Modern Science. Am J Clin Dermatol. 2018;19(1):103-17.

15. Vogel P, Machado IK, Garavaglia J, Zani VT, de Souza D, Dal Bosco SM. Polyphenols benefits of olive leaf (Olea europaea l) to human health. Nutr Hosp. 2015;31(3):1427-33.

16. Lockyer S, Yaqoob P, Spencer JPE, Rowland I. Olive leaf phenolics and cardiovascular risk reduction: Physiological effects and mechanisms of action. Nutr Aging. 2012;1(2):125-40.

17. Martiniakova M, Babikova M, Omelka R. PHARMACOLOGICAL AGENTS AND NATURAL COMPOUNDS: AVAILABLE TREATMENTS FOR OSTEOPOROSIS. Journal of Physiology and Pharmacology. 2020;71(3):307-20.

18. Nediani C, Ruzzolini J, Romani A, Calorini L. Oleuropein, a Bioactive Compound from Olea europaea L., as a Potential Preventive and Therapeutic Agent in Non-Communicable Diseases. 2019;8(12).

19. Saibandith B, Spencer JPE, Rowland IR, Commane DM. Olive Polyphenols and the Metabolic Syndrome. Molecules. 2017;22(7).

20. Kendall M, Batterham M, Obied H, Prenzler PD, Ryan D, Robards K. Zero effect of multiple dosage of olive leaf supplements on urinary biomarkers of oxidative stress in healthy humans. Nutrition (Burbank, Los Angeles County, Calif). 2009;25(3):270-80.

21. Ahmed KM. The effect of olive leaf extract in decreasing the expression of two pro-inflammatory cytokines in patients receiving chemotherapy for cancer. A randomized clinical trial. The Saudi dental journal. 2013;25(4):141-7.

22. Fernández-Aparicio Á, Perona JS, Castellano JM, Correa-Rodríguez M, Schmidt-Riovalle J, González-Jiménez E. Oleanolic acid-enriched olive oil alleviates the interleukin-6 overproduction induced by postprandial triglyceride-rich lipoproteins in thp-1 macrophages. Nutrients. 2021;13(10).

23. Somerville V, Moore R, Braakhuis A. The Effect of Olive Leaf Extract on Upper Respiratory Illness in High School Athletes: A Randomised Control Trial. Nutrients. 2019;11(2).

24. Alecci U, Bonina F, Bonina A, Rizza L, Inferrera S, Mannucci C, et al. Efficacy and Safety of a Natural Remedy for the Treatment of Gastroesophageal Reflux: A Double-Blinded Randomized-Controlled Study. Evidence-Based Complementary and Alternative Medicine. 2016;2016.

25. Caruso M, Frasca G, Di Giuseppe PL, Pennisi A, Tringali G, Bonina FP. Effects of a new nutraceutical ingredient on allergen-induced sulphidoleukotrienes production and CD63 expression in allergic subjects. International immunopharmacology. 2008;8(13-14):1781-6.

26. Elkafrawy N, Younes K, Naguib A, Badr H, Kamal Zewain S, Kamel M, et al. Antihypertensive efficacy and safety of a standardized herbal medicinal product of Hibiscus sabdariffa and Olea europaea extracts (NW Roselle): A phase-II, randomized, double-blind, captopril-controlled clinical trial. Phytother Res. 2020;34(12):3379-87.

27. Florentin M, Liberopoulos E, Elisaf MS, Tsimihodimos V. No effect of fenugreek, bergamot and olive leaf extract on glucose homeostasis in patients with prediabetes: a randomized double-blind placebo-controlled study. Archives of medical sciences Atherosclerotic diseases. 2019;4:e162-e6.

28. Macarro MS, Rodríguez JPM, Morell EB, Pérez-Piñero S, Victoria-Montesinos D, García-Muñoz AM, et al. Effect of a combination of citrus flavones and flavanones and olive polyphenols for the reduction of cardiovascular disease risk: An exploratory randomized, double-blind, placebo-controlled study in healthy subjects. Nutrients. 2020;12(5).

29. Mainini G, Passaro M, Schiattarella A, de Franciscis P, Di Donna MC, Trezza G. Prevention and treatment of cystitis during menopause: Efficacy of a nutraceutical containing D-mannose, inulin, cranberry, bearberry, Olea europaea, Orthosiphon and Lactobacillus acidophilus. Prz Menopauzalny. 2020;19(3):130-4.

30. Malfa GA, Di Giacomo C, Cardia L, Sorbara EE, Mannucci C, Calapai G. A standardized extract of Opuntia ficus-indica (L.) Mill and Olea europaea L. improves gastrointestinal discomfort: A double-blinded randomized-controlled study. 2021;35(7):3756-68.

31. Nobile V, Schiano I, Peral A, Giardina S, Spartà E, Caturla N. Antioxidant and reduced skin-ageing effects of a polyphenol-enriched dietary supplement in response to air pollution: a randomized, double-blind, placebo-controlled study. Food & nutrition research. 2021;65.

32. Victoria-Montesinos D, Ruiz MSA, Rubia ALJ, Martinez DG, Perez-Pinero S, Macarro MS, et al. Effectiveness of Consumption of a Combination of Citrus Fruit Flavonoids and Olive Leaf Polyphenols to Reduce Oxidation of Low-Density Lipoprotein in Treatment-Naive Cardiovascular Risk Subjects: A Randomized Double-Blind Controlled Study. Antioxidants. 2021;10(4).

33. Wong RHX, Garg ML, Wood LG, Howe PRC. Antihypertensive Potential of Combined Extracts of Olive Leaf, Green Coffee Bean and Beetroot: A Randomized, Double-Blind, Placebo-Controlled Crossover Trial. Nutrients. 2014;6(11):4881-94.

34. Dinu M, Pagliai G, Scavone F, Bellumori M, Cecchi L, Nediani C, et al. Effects of an Olive By-Product Called Pâté on Cardiovascular Risk Factors. Journal of the American College of Nutrition. 2021;40(7):617-23.

35. Kountouri AM, Mylona A, Kaliora AC, Andrikopoulos NK. Bioavailability of the phenolic compounds of the fruits (drupes) of Olea europaea (olives): impact on plasma antioxidant status in humans. Phytomedicine : international journal of phytotherapy and phytopharmacology. 2007;14(10):659-67.

36. Togni S, Maramaldi G, Conte C, Milano E, Giacomelli L. Photoprotective and antioxidant effects of a standardised olive (Olea europaea) extract in healthy volunteers. Esper Dermatol. 2015;17(4):143-8.

37. Alvarez-Cuesta E, Aragoneses-Gilsanz E, Martín-Garcia C, Berges-Gimeno P, Gonzalez-Mancebo E, Cuesta-Herranz J. Immunotherapy with depigmented glutaraldehyde-polymerized extracts: changes in quality of life. Clinical and experimental allergy : journal of the British Society for Allergy and Clinical Immunology. 2005;35(5):572-8.

38. Liccardi G, Baldi G, Berra A, Ciccarelli A, Cutajar M, D’Amato M, et al. Allergy in Urban elderly population living in campania region (Southern Italy). A multicenter study. Eur Ann Allergy Clinical Immunol. 2016;48(4):158-60.

39. Moreno C, De San Pedro B, Millan C, Panizo C, Martin S, Florido F. Exploratory study of tolerability and immunological effect of a short up-dosing immunotherapy phase with a standardised allergen extract derived from pollen of Olea europaea. Clinical and Translational Allergy. 2015;5.

40. Til-Pérez G, Carnevale C, Sarría-Echegaray PL, Arancibia-Tagle D, Chugo-Gordillo S, Tomás-Barberán MD. Sensitization profile in patients with respiratory allergic diseases: differences between conventional and molecular diagnosis (a cross-sectional study). Clinical and molecular allergy : CMA. 2019;17:8.

41. Olivieri F, Montesanto A, Malatesta G, Di Pillo R, Antonicelli R, Arslan K, et al. Protoscolicidal effect of oleuropein: an in vitro study. Trials. 2019;35(1):30-4.

42. Sari HA, Ekinci R. The effect of ultrasound application and addition of leaves in the malaxation of olive oil extraction on the olive oil yield, oxidative stability and organoleptic quality. Food Science and Technology. 2017;37(3):493-9.

43. Tajalli F. Comparison of polyphenolic compounds concentration and antiradical activity between grape and currant seeds and olive (olea europaea) pits extracts. Biotechnol An Indian J. 2014;9(11):435-9.

44. Cabrera-Vique C, Navarro-Alarcón M, Martínez CR, Fonollá-Joya J. Hypotensive effect of an extract of bioactive compounds of olive leaves: Preliminary clinical study. Nutr Hosp. 2015;32(1):242-9.

45. de Bock M, Thorstensen EB, Derraik JG, Henderson HV, Hofman PL, Cutfield WS. Human absorption and metabolism of oleuropein and hydroxytyrosol ingested as olive (Olea europaea L.) leaf extract. Molecular nutrition & food research. 2013;57(11):2079-85.

46. Hermans MP, Lempereur P, Salembier JP, Maes N, Albert A, Jansen O, et al. Supplementation Effect of a Combination of Olive (Olea europea L.) Leaf and Fruit Extracts in the Clinical Management of Hypertension and Metabolic Syndrome. Antioxidants (Basel, Switzerland). 2020;9(9).

47. Tenore GC, Caruso D, D’avino M, Buonomo G, Caruso G, Ciampaglia R, et al. A pilot screening of agro-food waste products as sources of nutraceutical formulations to improve simulated postprandial glycaemia and insulinaemia in healthy subjects. Nutrients. 2020;12(5).

48. Wanitphakdeedecha R, Ng JNC, Junsuwan N, Phaitoonwattanakij S, Phothong W, Eimpunth S, et al. Efficacy of olive leaf extract–containing cream for facial rejuvenation: A pilot study. J Cosmet Dermatol. 2020;19(7):1662-6.

49. Zam W AA. Olive leaves herbal tea effect in type 2 diabetic patients with prehypertension. European Journal of Pharmaceutical and Medical Research. 2017.

50. Kendall M, Batterham M, Callahan DL, Jardine D, Prenzler PD, Robards K, et al. Randomized controlled study of the urinary excretion of biophenols following acute and chronic intake of olive leaf supplements. Food Chemistry. 2012;130(3):651-9.

51. Pyner A, Chan SY, Tumova S, Kerimi A, Williamson G. Indirect Chronic Effects of an Oleuropein-Rich Olive Leaf Extract on Sucrase-Isomaltase In Vitro and In Vivo. Nutrients. 2019;11(7).

52. Schulz V. Is olive leaf extract effective against hypertension? A double-blind study in comparison with low-dose captopril. Z Phytother. 2011;32(3):128-9.
