# Supplementary material for: The effects of olive leaf extract on cardiovascular risk factors in the general adult population: a systematic review and meta-analysis of randomized controlled trials
Source: Diabetol Metab Syndr. 2022 Oct 21;14:151. doi: 10.1186/s13098-022-00920-y (PMC9585795; doi:10.1186/s13098-022-00920-y)
Supplement: Supplementary file 3 — Additional file 3: Study quality and risk of bias assessment using Cochrane collaboration tool. [file 13098_2022_920_MOESM3_ESM.docx]

**Additional file 3: Study quality and risk of bias assessment using Cochrane collaboration** **tool** ^a^

| **Author, Year** | **Randomization Process** | **Deviations from the intended intervention** | **Missing outcome data** | **Measurement of the outcomes** | **Selection of the reported results** | **Overall quality ^b^** |
| --- | --- | --- | --- | --- | --- | --- |
| Araki, 2018 | 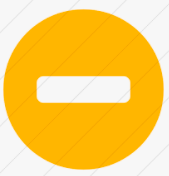 | 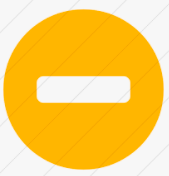 | 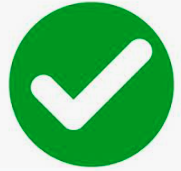 | 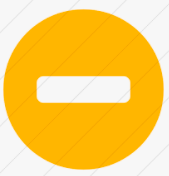 | 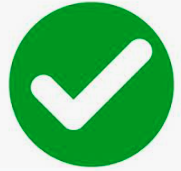 | 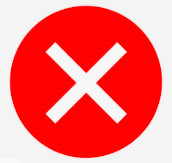 |
| Araki, 2019 | 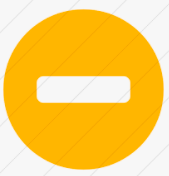 | 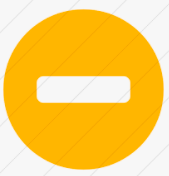 | 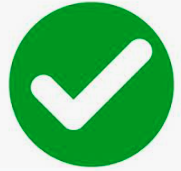 | 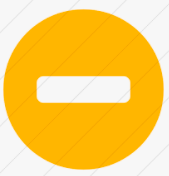 | 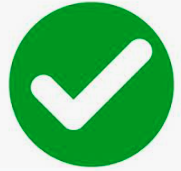 | 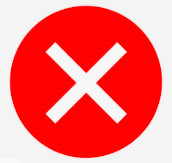 |
| de Bock, 2013 | 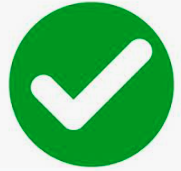 | 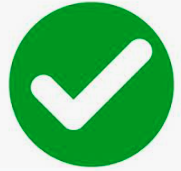 | 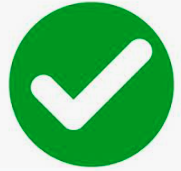 | 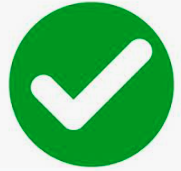 | 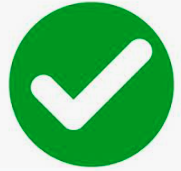 | 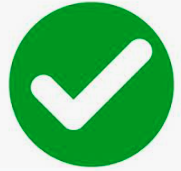 |
| Filip, 2015 | 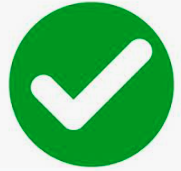 | 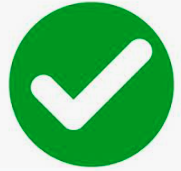 | 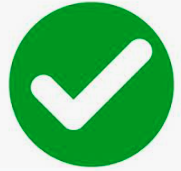 | 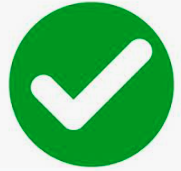 | 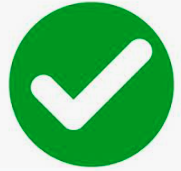 | 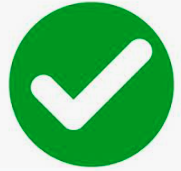 |
| Javadi, 2019 | 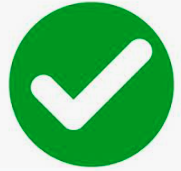 | 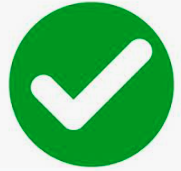 | 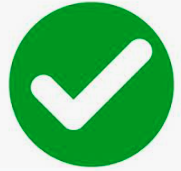 | 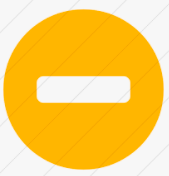 | 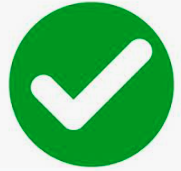 | 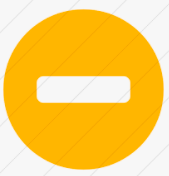 |
| Lockyer, 2017 | 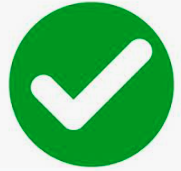 | 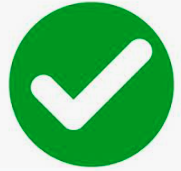 | 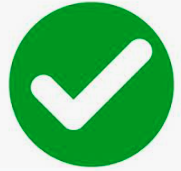 | 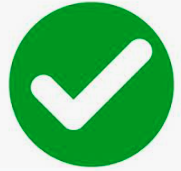 | 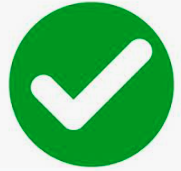 | 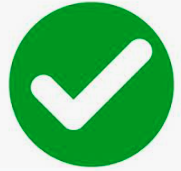 |
| Perrinjaquet-Moccetti, 2008 | 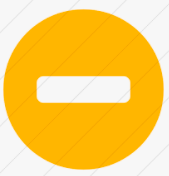 | 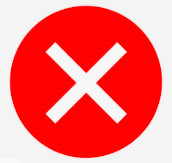 | 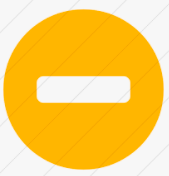 | 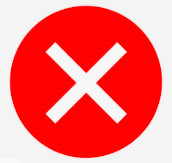 | 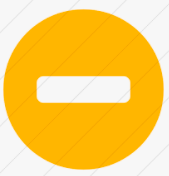 | 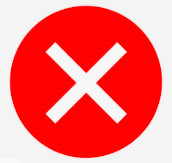 |
| Saberi, 2018 | 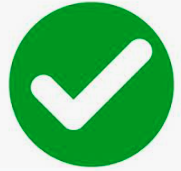 | 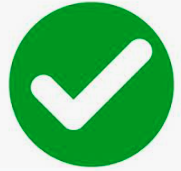 | 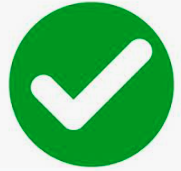 | 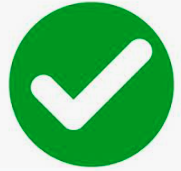 | 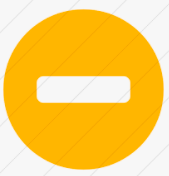 | 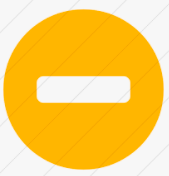 |
| Stevense, 2021 | 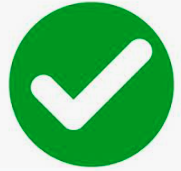 | 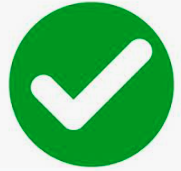 | 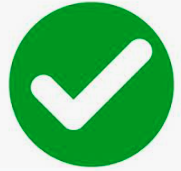 | 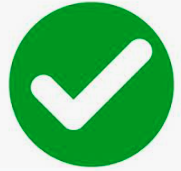 | 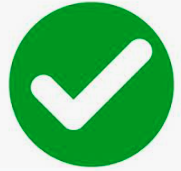 | 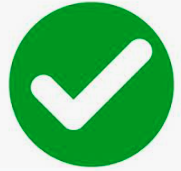 |
| Susalit, 2011 | 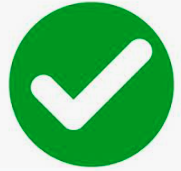 | 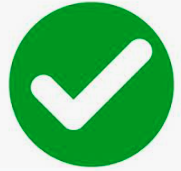 | 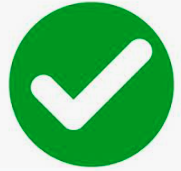 | 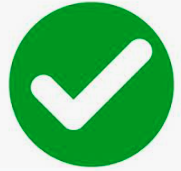 | 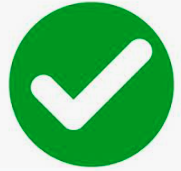 | 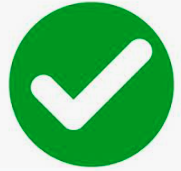 |
| Wainstein, 2012 | 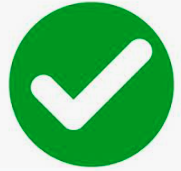 | 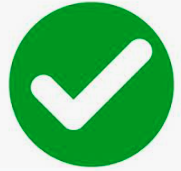 | 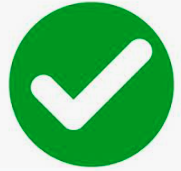 | 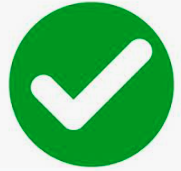 | 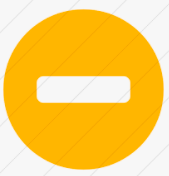 | 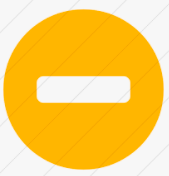 |
| Yaghoobzadeh, 2020 | 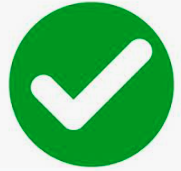 | 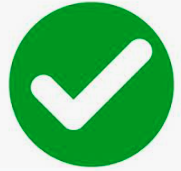 | 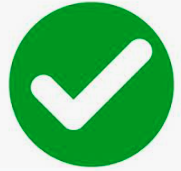 | 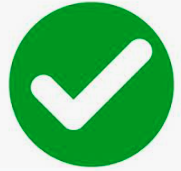 | 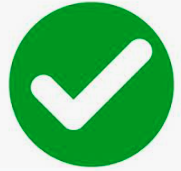 | 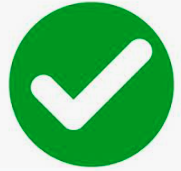 |

**^a^** Cochrane risk of Bias 2 (RoB2) (2019) was used for quality assessment.

^b^ Signs indicate high risk of bias (
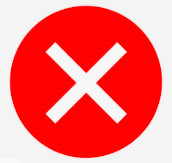
), some concerns (
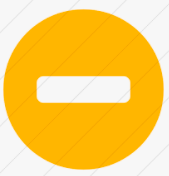
) and low risk of bias (
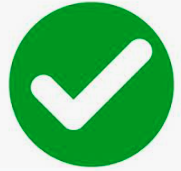
).

GRADE assessment of confidence in estimates of effect in randomized trials

| **Outcome** | **Studies/ Participants** | **Risk of bias** | **Inconsistency** | **Indirectness** | **Imprecision** | **Publication bias** | **Certainty** |
| --- | --- | --- | --- | --- | --- | --- | --- |
| ***Glucose homeostasis*** | | | | | | | |
| FBS (mg/dl) | 6/ 485 | Not serious^1^ | Not serious (I^2^=0) | Not serious | Serious^2^ | NA | ⨁⨁⨁◯ Moderate |
| Insulin (μU/L) | 4/ 320 | Not serious | Serious^3^ | Serious^4^ | Serious^2^ | NA | ⨁⨁◯◯ Low |
| HbA1c (%) | 3/ 175 | Serious^5^ | Serious^6^ | Serious^4^ | Serious^2^ | NA | ⨁◯◯◯ Very low |
| HOMA-IR | 3/ 221 | Serious^7^ | Serious^8^ | Serious^4^ | Serious^2^ | NA |  |
| ***Lipid profile*** | | | | | | | |
| LDL-c (mg/dl) | 9/ 625 | Not serious | Not serious (I^2^=56.3)^9^ | Not serious | Serious^2^ | NA | ⨁⨁⨁◯ Moderate |
| HDL-c (mg/dl) | 9/ 646 | Not serious | Not serious (I^2^=54.2)^10^ | Not serious | Serious^2^ | NA | ⨁⨁⨁◯ Moderate |
| TG (mg/dl) | 7/ 567 | Not serious | Not serious (I^2^=68.7)^11^ | Not serious | Serious^2^ | NA | ⨁⨁⨁◯ Moderate |
| TC (mg/dl) | 7/ 549 | Not serious | Not serious (I^2^=65.6)^12^ | Not serious | Serious^2^ | NA | ⨁⨁⨁◯ Moderate |
| ***Blood pressure*** | | | | | | | |
| SBP (mmHg) | 6/ 401 | Not serious | Not serious (I^2^=19.9) | Not serious | Serious^2^ | NA | ⨁⨁⨁◯ Moderate |
| DBP (mmHg) | 6/ 401 | Not serious | Not serious (I^2^=47.5) | Not serious | Serious^2^ | NA | ⨁⨁⨁◯ Moderate |
| ***Liver & Kidney*** | | | | | | | |
| ALT (U/L) | 3/ 315 | Not serious | Not serious (I^2^=0) | Serious^4^ | Serious^2^ | NA | ⨁⨁◯◯ Low |
| AST (U/L) | 3/ 315 | Not serious | Not serious (I^2^=0) | Serious^4^ | Serious^2^ | NA | ⨁⨁◯◯ Low |
| ALP (U/L) | 3/ 184 | Not serious | Not serious (I^2^=0) | Serious^4^ | Serious^2^ | NA | ⨁⨁◯◯ Low |
| Creatinine | 3/ 285 | Not serious | Not serious (I^2^=0) | Serious^4^ | Serious^2^ | NA | ⨁⨁◯◯ Low |
| ***Inflammatory markers*** | | | | | | | |
| Hs-CRP | 3/ 238 | Not serious | Serious^13^ | Serious^4^ | Serious^2^ | NA | ⨁◯◯◯ Very low |
| TNF-α | 3/ 186 | Not serious | Serious^14^ | Serious^4^ | Serious^2^ | NA | ⨁◯◯◯ Very low |
| IL-6 | 4/ 220 | Not serious | Serious^15^ | Serious^4^ | Serious^2^ | NA | ⨁◯◯◯ Very low |
| IL-8 | 3/ 188 | Not serious | Serious^16^ | Serious^4^ | Serious^2^ | NA | ⨁◯◯◯ Very low |
| LDL-ox | 3/ 267 | Not serious | Not serious (I^2^=0) | Serious^4^ | Serious^2^ | NA | ⨁⨁◯◯ Low |
| ALP, Alkaline phosphatase; ALT, Alanine transaminase; AST, Aspartate transaminase; DBP, Diastolic blood pressure; FBS, fasting blood sugar; HbA1c, Hemoglobin A1C; HDL, High-density lipoprotein; HOMA-IR, Homeostatic model assessment for insulin resistance; hs-CRP, high-sensitivity C-reactive protein; IL-6, Interleukin 6; IL-8, Interleukin 8; LDL, Low-density lipoprotein; NA, Not assessed; SBP, Systolic blood pressure; TC, Total cholesterol; TG, Triglyceride; TNF, Tumor necrosis factor.   1. The half of studies (3 of 6) were at low and unclear quality, however the direction and significance of effect sizes did not different between overall analysis vs. high quality trials category (Effect size for good quality trails (WMD= -0.90; 95% CI: -0.42.660, 0.86); n=3). Not downgraded. 2. The sample size did not reach the optimal information size (N ≤ 800). Downgraded. 3. I^2^=54.4, P heterogeneity= 0.09. The sensitivity analysis did not explain the source of heterogeneity. Downgraded. 4. The number of eligible studies is equal or less than five. Downgraded. 5. All of studies were at poor and fair quality. Main trial limitations were lack of random sequence generation, allocation concealment, and personnel and blinding of outcome assessment. Downgraded. 6. I^2^=78.2, P heterogeneity= 0.08. The sensitivity analysis did not explain the source of heterogeneity. Downgraded. 7. Most of studies were at poor and fair quality. Main trial limitations were lack of random sequence generation, allocation concealment, and personnel and blinding of outcome assessment. Downgraded. 8. I^2^=53.4, P heterogeneity= 0.12. The sensitivity analysis did not explain the source of heterogeneity. Downgraded. 9. I^2^=56.3, P heterogeneity=0.03. The subgroup based on study duration explained the source of heterogeneity. (Short duration: -4.01; 95% CI: -7.49, -0.53, I^2^=0, P heterogeneity=0.48). Not downgraded. 10. I^2^=54.2, P heterogeneity=0.03. The subgroup based on study duration explained the source of heterogeneity. (Short duration: -0.08; 95% CI: -1.39, 1.23, I^2^=0, P heterogeneity=0.61). Not downgraded. 11. I^2^=68.7, P heterogeneity=0.06. The subgroup based on study design explained the source of heterogeneity. (Parallel design: -10.42; 95% CI: -18.68, -2.15, I^2^=0, P heterogeneity=0.77). Not downgraded 12. I^2^=65.6, P heterogeneity=0.008. The subgroup based on study design explained the source of heterogeneity. (Parallel design: -4.60; 95% CI: -9.51, 0.30, I^2^=12.8, P heterogeneity=0.33). Not downgraded. 13. I^2^=63.9, P heterogeneity= 0.006. The sensitivity analysis did not explain the source of heterogeneity. Downgraded. 14. I^2^=75.1, P heterogeneity= 0.018. The sensitivity analysis did not explain the source of heterogeneity. Downgraded. 15. I^2^=58.1, P heterogeneity= 0.07. The sensitivity analysis did not explain the source of heterogeneity. Downgraded. 16. I^2^=82.7, P heterogeneity= 0.003. The sensitivity analysis did not explain the source of heterogeneity. Downgraded. | | | | | | | |
