# Supplementary material for: The effects of olive leaf extract on cardiovascular risk factors in the general adult population: a systematic review and meta-analysis of randomized controlled trials
Source: Diabetol Metab Syndr. 2022 Oct 21;14:151. doi: 10.1186/s13098-022-00920-y (PMC9585795; doi:10.1186/s13098-022-00920-y)
Supplement: Supplementary file 4 — Additional file 4: Meta-analysis showing the effect of OLE supplementation on total cholesterol based on several subgroups. [file 13098_2022_920_MOESM4_ESM.docx]

| **Additional file 4: Meta-analysis showing the eﬀect of OLE supplementation on total cholesterol (mg/dl) based on several subgroups** | | | | | | | |
| --- | --- | --- | --- | --- | --- | --- | --- |
| **TC (mg/dl)** | **Studies (n)** | **Meta-analysis** | | **Heterogeneity** | | | |
|  |  | **WMD (95%CI)** | **P effect** | **Q statistic** | **I^2^ (%)** | **P within group** | **P between group** |
| **Sex** |  |  |  |  |  |  | 0.65 |
| Male | 2 | -3.73 (-20.40, 12.94) | 0.66 | 12.29 | 91.9 | <0.001 |  |
| Female | 1 | 0.00 (-17.92, 17.92) | 1.00 | 0 | - | - |  |
| Both | 4 | -4.96 (-10.82, 0.90) | 0.10 | 4.31 | 30.4 | 0.23 |  |
| **Study design** |  |  |  |  |  |  | 0.45 |
| Cross-over | 2 | -3.73 (-20.40, 12.94) | 0.66 | 12.29 | 91.9 | <0.001 |  |
| Parallel | 5 | -4.60 (-9.51, 0.30) | 0.07 | 4.58 | 12.8 | 0.33 |  |
| **Study duration** |  |  |  |  |  |  | 0.22 |
| < 8 weeks | 4 | -4.67 (-10.93, 1.29) | 0.14 | 6.25 | 52 | 0.10 |  |
| ≥ 8 weeks | 6 | -2.05 (-7.86, 3.76) | 0.49 | 10.61 | 52.9 | 0.06 |  |
| **BMI** |  |  |  |  |  |  | 0.13 |
| Normal-weight | 4 | -6.69 (-11.90, -1.49) | 0.01 | 2.65 | 0 | 0.45 |  |
| Obese | 3 | -2.46 (-12.65, 7.73) | 0.64 | 12.52 | 84 | 0.002 |  |
| **Hypertension** |  |  |  |  |  |  | <0.001 |
| Yes | 4 | -9.14 (-13.80, -4.47) | <0.001 | 3.24 | 7.5 | 0.36 |  |
| No | 3 | 2.73 (-1.89, 7.35) | 0.25 | 0.94 | 0 | 0.63 |  |
| **Dyslipidemia** |  |  |  |  |  |  | 0.34 |
| Yes | 1 | 0.00 (-7.87, 7.87) | 1.00 | 0 | - | - |  |
| No | 6 | -4.74 (-11.95, 2.48) | 0.20 | 16.54 | 69.8 | 0.005 |  |

| Meta-analysis showing the eﬀect of OLE supplementation on LDL cholesterol (mg/dl) based on several subgroups | | | | | | | |
| --- | --- | --- | --- | --- | --- | --- | --- |
| **LDL-c (mg/dl)** | **Studies (n)** | **Meta-analysis** | | **Heterogeneity** | | | |
|  |  | **WMD (95%CI)** | **P effect** | **Q statistic** | **I^2^ (%)** | **P within group** | **P between group** |
| **Sex** |  |  |  |  |  |  | 0.43 |
| Male | 2 | -2.87 (-11.59, 5.847) | 0.52 | 4.52 | 77.9 | 0.03 |  |
| Female | 1 | 9.29 (-8.60, 27.18) | 0.31 | 0 | - | - |  |
| Both | 6 | -1.19 (-6.30, 3.91) | 0.65 | 11.05 | 54.8 | 0.050 |  |
| **Study design** |  |  |  |  |  |  | 0.60 |
| Cross-over | 2 | -2.87 (-11.59, 5.84) | 0.52 | 4.52 | 77.9 | 0.03 |  |
| Parallel | 7 | -0.55 (-5.52, 4.43) | 0.83 | 12.47 | 51.9 | 0.052 |  |
| **Study duration** |  |  |  |  |  |  | 0.15 |
| < 8 weeks | 4 | -4.01 (-7.49, -0.53) | 0.02 | 2.49 | 0 | 0.48 |  |
| ≥ 8 weeks | 8 | -0.26 (-4.37, 3.85) | 0.90 | 13.25 | 47.2 | 0.07 |  |
| **BMI** |  |  |  |  |  |  | 0.62 |
| Normal weight | 5 | 0.18 (-5.27, 5.64) | 0.95 | 7.22 | 44.6 | 0.12 |  |
| Obese | 4 | -2.96 (-9.54, 3.63) | 0.38 | 9.79 | 69.4 | 0.02 |  |
| **Hypertension** |  |  |  |  |  |  | 0.01 |
| Yes | 4 | -4.60 (-8.26, -0.94) | 0.014 | 3.40 | 11.7 | 0.33 |  |
| No | 5 | 1.35 (-4.37, 7.07) | 0.64 | 7.91 | 49.4 | 0.09 |  |
| **Dyslipidemia** |  |  |  |  |  |  | 0.01 |
| Yes | 2 | 4.74 (-1.15, 10.63) | 0.11 | 0.09 | 0 | 0.76 |  |
| No | 7 | -3.14 (-7.26, 0.97) | 0.13 | 11.18 | 46.3 | 0.08 |  |

| Meta-analysis showing the eﬀect of OLE supplementation on HDL cholesterol (mg/dl) based on several subgroups | | | | | | | |
| --- | --- | --- | --- | --- | --- | --- | --- |
| **HDL-c (mg/dl)** | **Studies (n)** | **Meta-analysis** | | **Heterogeneity** | | | |
|  |  | **WMD (95%CI)** | **P effect** | **Q statistic** | **I^2^ (%)** | **P within group** | **P between group** |
| **Sex** |  |  |  |  |  |  | 0.002 |
| Male | 2 | -1.24 (-2.42, -0.07) | 0.04 | 0.07 | 0 | 0.79 |  |
| Female | 1 | 4.34 (0.78, 7.90) | 0.02 | 0 | - | - |  |
| Both | 6 | 1.02 (-0.23, 2.27) | 0.11 | 4.86 | 0 | 0.43 |  |
| **Study design** |  |  |  |  |  |  | 0.002 |
| Cross-over | 2 | -1.24 (-2.42, -0.07) | 0.04 | 0.07 | 0 | 0.79 |  |
| Parallel | 7 | 1.35 (-0.14, 2.84) | 0.08 | 7.83 | 23.3 | 0.25 |  |
| **Study duration** |  |  |  |  |  |  | 0.67 |
| < 8 weeks | 4 | -0.08 (-1.39, 1.23) | 0.91 | 1.80 | 0 | 0.61 |  |
| ≥ 8 weeks | 8 | 0.69 (-0.92, 2.31) | 0.40 | 15.73 | 55.5 | 0.03 |  |
| **BMI** |  |  |  |  |  |  | 0.003 |
| Normal weight | 5 | 1.51 (-0.51, 3.54) | 0.14 | 7.35 | 45.6 | 0.12 |  |
| Obese | 4 | -1.03 (-2.13, 0.06) | 0.06 | 1.05 | 0 | 0.79 |  |
| **Hypertension** |  |  |  |  |  |  | 0.13 |
| Yes | 4 | 0.71 (-1.17, 2.59) | 0.46 | 5.52 | 45.6 | 0.14 |  |
| No | 5 | 0.12 (-2.28, 2.53) | 0.92 | 9.64 | 58.5 | 0.05 |  |
| **Dyslipidemia** |  |  |  |  |  |  | 0.25 |
| Yes | 2 | -2.21 (-6.22, 1.80) | 0.28 | 0.57 | 0 | 0.45 |  |
| No | 7 | 0.67 (-0.91, 2.24) | 0.41 | 15.61 | 61.6 | 0.02 |  |

| Meta-analysis showing the eﬀect of OLE supplementation on triglyceride (mg/dl) based on several subgroups | | | | | | | |
| --- | --- | --- | --- | --- | --- | --- | --- |
| **TG (mg/dl)** | **Studies (n)** | **Meta-analysis** | | **Heterogeneity** | | | |
|  |  | **WMD (95%CI)** | **P effect** | **Q statistic** | **I^2^ (%)** | **P within group** | **P between group** |
| **Sex** |  |  |  |  |  |  | 0.34 |
| Male | 2 | -6.77 (-25.83, 12.29) | 0.49 | 16.03 | 93.8 | <0.001 |  |
| Female | 1 | -23.05 (-51.087, 5.77) | 0.12 | 0 | - | - |  |
| Both | 4 | -9.29 (-17.91, -0.66) | 0.03 | 0.98 | 0 | 0.805 |  |
| **Study design** |  |  |  |  |  |  | 0.25 |
| Cross-over | 2 | -6.77 (-25.83, 12.29) | 0.47 | 16.03 | 93.8 | <0.001 |  |
| Parallel | 5 | -10.42 (-18.68, -2.15) | 0.01 | 1.79 | 0 | 0.77 |  |
| **Study duration** |  |  |  |  |  |  | <0.001 |
| < 8 weeks | 3 | -13.88 (-20.11, -7.66) | <0.001 | 3.04 | 34.2 | 0.22 |  |
| ≥ 8 weeks | 6 | -5.88 (-13.49, 1.72) | 0.13 | 6.57 | 23.9 | 0.25 |  |
| **BMI** |  |  |  |  |  |  | 0.18 |
| Normal weight | 4 | -9.21 (-18.14, -0.29) | 0.04 | 1.29 | 0 | 0.73 |  |
| Obese | 3 | -9.31 (-24.38, 5.76) | 0.23 | 16.07 | 87.6 | <0.001 |  |
| **Hypertension** |  |  |  |  |  |  | 0.001 |
| Yes | 3 | -14.32 (-19.36, -9.28) | <0.001 | 2.51 | 20.2 | 0.28 |  |
| No | 4 | -7.20 (-19.87, 5.46) | 0.26 | 5.63 | 46.7 | 0.13 |  |
| **Dyslipidemia** |  |  |  |  |  |  | 0.68 |
| Yes | 2 | -12.12 (-26.88, 2.64) | 0.11 | 0.46 | 0 | 0.50 |  |
| No | 5 | -8.82 (-19.15, 1.50) | 0.09 | 18.53 | 78.4 | 0.001 |  |

| Meta-analysis showing the eﬀect of OLE supplementation on fasting blood glucose (mg/dl) based on several subgroups | | | | | | | |
| --- | --- | --- | --- | --- | --- | --- | --- |
| **FBS (mg/dl)** | **Studies (n)** | **Meta-analysis** | | **Heterogeneity** | | | |
|  |  | **WMD (95%CI)** | **P effect** | **Q statistic** | **I^2^ (%)** | **P within group** | **P between group** |
| **Sex** |  |  |  |  |  |  | 0.64 |
| Male | 1 | -1.80 (-4.35, 0.75) | 0.17 | 0 | - | - |  |
| Both | 5 | -1.01 (-2.88, 0.86) | 0.29 | 4.67 | 14.3 | 0.32 |  |
| **Study design** |  |  |  |  |  |  | 0.64 |
| Cross-over | 1 | -1.80 (-4.35, 0.75) | 0.17 | 0 | - | - |  |
| Parallel | 5 | -1.01 (-2.88, 0.86) | 0.29 | 4.67 | 14.3 | 0.32 |  |
| **Study duration** |  |  |  |  |  |  | 0.88 |
| < 8 weeks | 2 | -0.41 (-3.84, 3.02) | 0.81 | 2.04 | 50.9 | 0.15 |  |
| ≥ 8 weeks | 4 | -1.01 (-2.88, 0.86) | 0.29 | 4.67 | 14.3 | 0.32 |  |
| **BMI** |  |  |  |  |  |  | 0.54 |
| Normal weight | 3 | -0.76 (-2.96, 1.44) | 0.50 | 0.60 | 0 | 0.74 |  |
| Obese | 3 | -1.49 (-4.20, 1.21) | 0.28 | 3.92 | 48.9 | 0.14 |  |
| **Hypertension** |  |  |  |  |  |  | 0.86 |
| Yes | 3 | -1.19 (-3.00, 0.62) | 0.20 | 0.96 | 0 | 0.62 |  |
| No | 3 | -1.25 (-4.44, 1.94) | 0.44 | 3.90 | 48.7 | 0.14 |  |
| **Dyslipidemia** |  |  |  |  |  |  | 0.25 |
| Yes | 2 | 0.25 (-2.79, 3.29) | 0.87 | 1.03 | 2.7 | 0.31 |  |
| No | 4 | -1.73 (-3.33, -0.13) | 0.03 | 2.55 | 0 | 0.47 |  |

| Meta-analysis showing the eﬀect of OLE supplementation on systolic blood pressure (mmHg) based on several subgroups | | | | | | | |
| --- | --- | --- | --- | --- | --- | --- | --- |
| **SBP (mmHg)** | **Studies (n)** | **Meta-analysis** | | **Heterogeneity** | | | |
|  |  | **WMD (95%CI)** | **P effect** | **Q statistic** | **I^2^ (%)** | **P within group** | **P between group** |
| **Sex** |  |  |  |  |  |  | 0.27 |
| Male | 2 | -2.49 (-5.89, 0.91) | 0.15 | 1.13 | 11.5 | 0.29 |  |
| Both | 4 | -5.27 (-9.04, -1.50) | 0.006 | 3.92 | 23.4 | 0.27 |  |
| **Study design** |  |  |  |  |  |  | 0.27 |
| Cross-over | 2 | -2.49 (-5.89, 0.91) | 0.15 | 1.13 | 11.5 | 0.29 |  |
| Parallel | 4 | -5.27 (-9.04, -1.50) | 0.006 | 3.92 | 23.4 | 0.27 |  |
| **Study duration** |  |  |  |  |  |  | 0.20 |
| < 8 weeks | 3 | -1.67 (-5.03, 1.70) | 0.33 | 2.64 | 24.3 | 0.27 |  |
| ≥ 8 weeks | 4 | -3.41 (-7.00, 0.17) | 0.06 | 3.98 | 24.6 | 0.26 |  |
| **BMI** |  |  |  |  |  |  | 0.05 |
| Normal weight | 3 | -7.05 (-10.94, -3.16) | <0.001 | 0.89 | 0 | 0.64 |  |
| Obese | 3 | -2.31 (-4.90, 0.27) | 0.08 | 1.40 | 0 | 0.50 |  |
| **Hypertension** |  |  |  |  |  |  | 0.08 |
| Yes | 4 | -4.81 (-7.27, -2.35) | <0.001 | 3.01 | 0.3 | 0.39 |  |
| No | 2 | -0.25 (-4.75, 4.24) | 0.91 | 0.19 | 0 | 0.66 |  |
| **Dyslipidemia** |  |  |  |  |  |  | 0.029 |
| Yes | 1 | -1.00 (-6.60, 4.60) | 0.73 | 0 | - | - |  |
| No | 5 | -4.47 (-7.39, -1.56) | 0.003 | 5.14 | 22.2 | 0.27 |  |

| Meta-analysis showing the eﬀect of OLE supplementation on diastolic blood pressure (mmHg) based on several subgroups | | | | | | | |
| --- | --- | --- | --- | --- | --- | --- | --- |
| **DBP (mmHg)** | **Studies (n)** | **Meta-analysis** | | **Heterogeneity** | | | |
|  |  | **WMD (95%CI)** | **P effect** | **Q statistic** | **I^2^ (%)** | **P within group** | **P between group** |
| **Sex** |  |  |  |  |  |  | 0.16 |
| Male | 2 | -0.47 (-4.21, 3.27) | 0.81 | 6.49 | 84.6 | 0.01 |  |
| Both | 4 | -2.14 (-4.41, 0.13) | 0.06 | 1.11 | 0 | 0.77 |  |
| **Study design** |  |  |  |  |  |  | 0.16 |
| Cross-over | 2 | -0.47 (-4.21, 3.27) | 0.81 | 6.49 | 84.6 | 0.01 |  |
| Parallel | 4 | -2.14 (-4.41, 0.13) | 0.06 | 1.11 | 0 | 0.77 |  |
| **Study duration** |  |  |  |  |  |  | 0.06 |
| < 8 weeks | 3 | -1.60 (-3.49, 0.28) | 0.09 | 1.92 | 0 | 0.38 |  |
| ≥ 8 weeks | 4 | 0.58 (-1.02, 2.17) | 0.48 | 2.42 | 0 | 0.49 |  |
| **BMI** |  |  |  |  |  |  | 0.14 |
| Normal weight | 3 | -2.48 (-5.07, 0.10) | 0.06 | 0.82 | 0 | 0.66 |  |
| Obese | 3 | -0.56 (-3.37, 2.25) | 0.69 | 6.58 | 69.6 | 0.04 |  |
| **Hypertension** |  |  |  |  |  |  | 0.005 |
| Yes | 4 | -2.45 (-4.13, -0.76) | 0.004 | 0.83 | 0 | 0.84 |  |
| No | 2 | 1.06 (-0.72, 2.84) | 0.24 | 0.85 | 0 | 0.36 |  |
| **Dyslipidemia** |  |  |  |  |  |  | 0.93 |
| Yes | 1 | -1.00 (-5.74, 3.74) | 0.68 | 0 | - | - |  |
| No | 5 | -1.26 (-3.47, 0.95) | 0.26 | 9.52 | 58 | 0.05 |  |
