# Supplementary material for: The effects of olive leaf extract on cardiovascular risk factors in the general adult population: a systematic review and meta-analysis of randomized controlled trials
Source: Diabetol Metab Syndr. 2022 Oct 21;14:151. doi: 10.1186/s13098-022-00920-y (PMC9585795; doi:10.1186/s13098-022-00920-y)
Supplement: Supplementary file 5 — Additional file 5: Forest plot of randomized controlled trials illustrating weighted mean differences in liver enzymes and creatinine. [file 13098_2022_920_MOESM5_ESM.docx]

**Additional file 5: Forest plot of randomized controlled trials illustrating weighted mean differences in liver enzymes and creatinine**

**
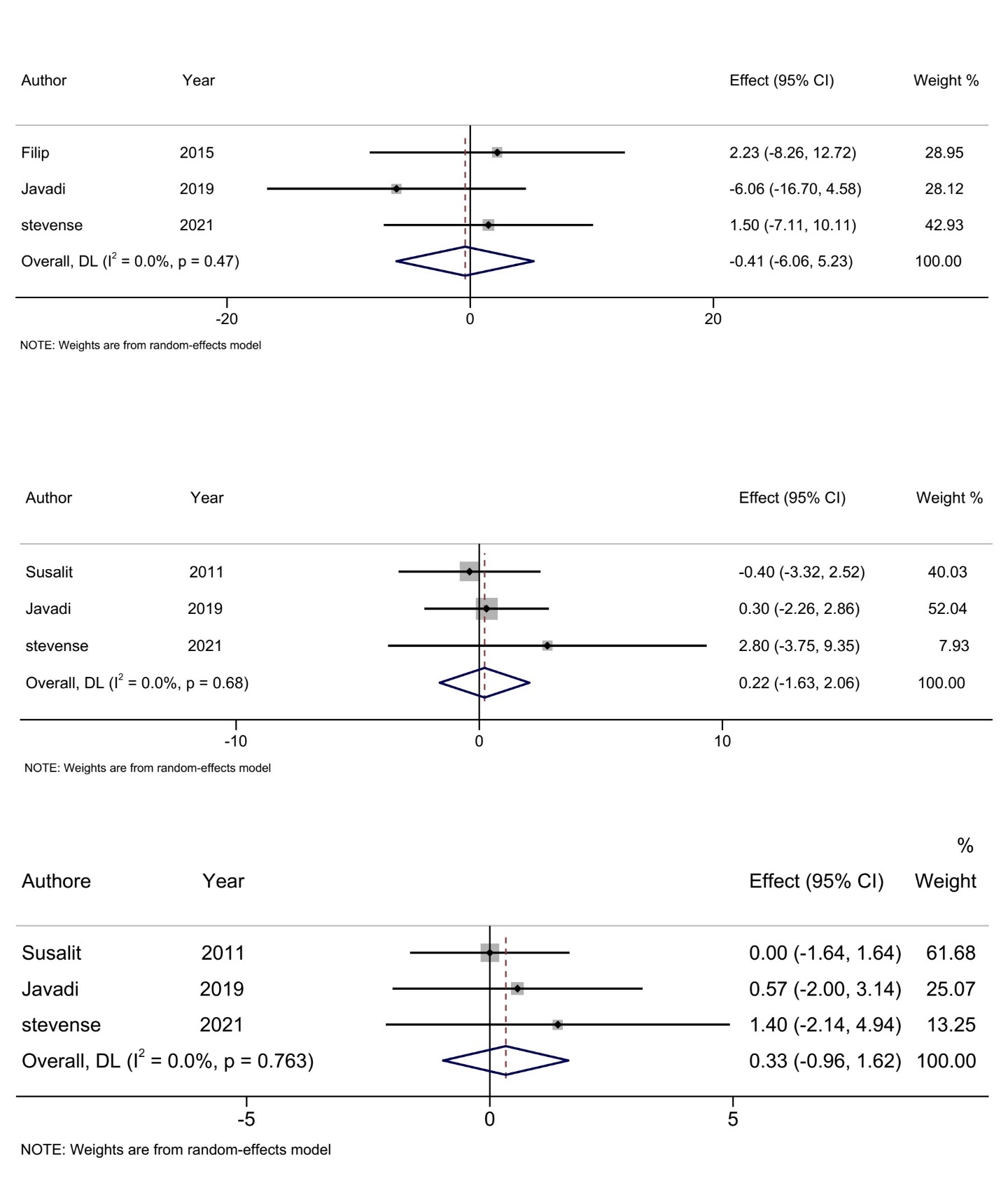
**

**C**

**B**

**A**

Forest plot of randomized controlled trials (RCTs) illustrating weighted mean differences in (a) ALP, (b) AST, and (c) ALT between OLE supplementation and control group for all eligible studies. Analysis was conducted using a random effect

**
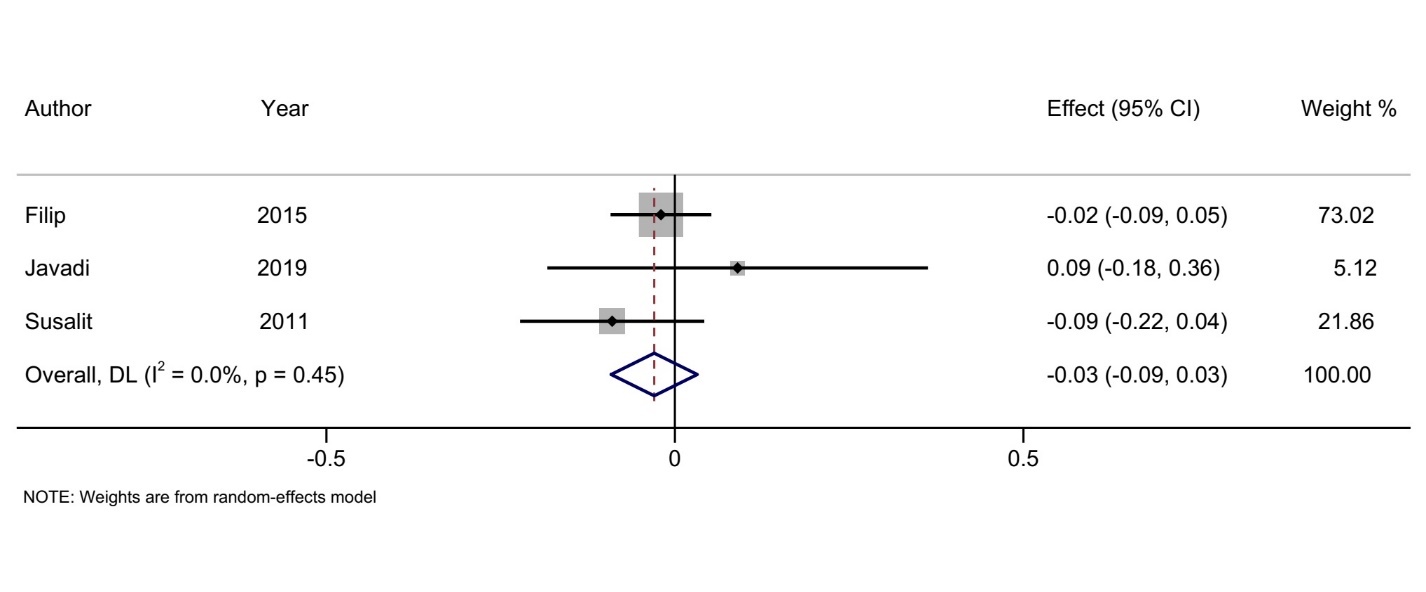
**

Forest plot of randomized controlled trials (RCTs) illustrating weighted mean differences in creatinine between OLE supplementation and control group for all eligible studies. Analysis was conducted using a random effect

**B**

**A**

**
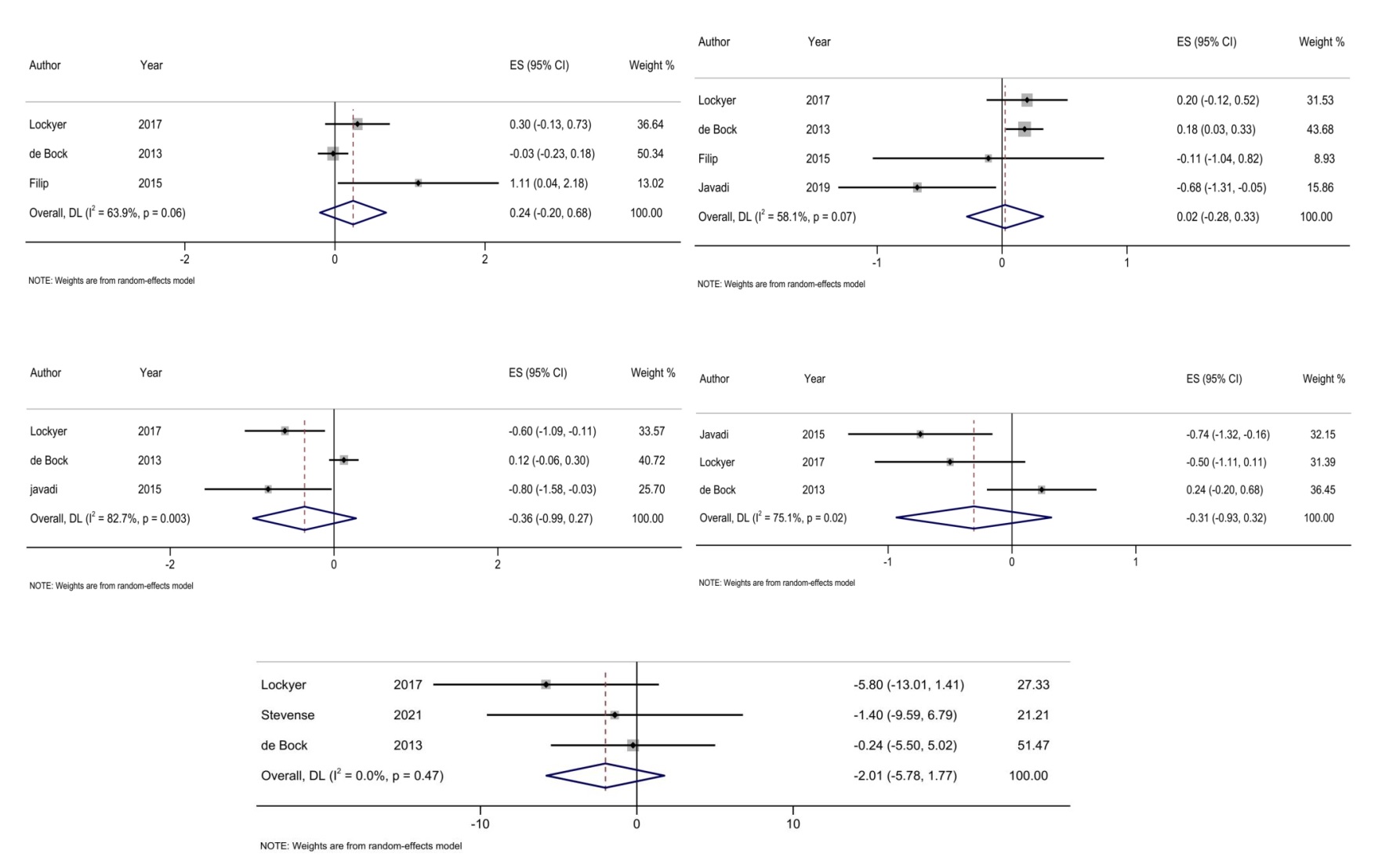
**

**C**

**D**

**E**

Forest plot of randomized controlled trials (RCTs) illustrating weighted mean differences in (a) hs-CRP, (b) IL-6, (c) IL-8, (d) TNF-α, and (e) LDL-ox between OLE supplementation and control group for all eligible studies. Analysis was conducted using a random effect
