# Supplementary material for: The effects of olive leaf extract on cardiovascular risk factors in the general adult population: a systematic review and meta-analysis of randomized controlled trials
Source: Diabetol Metab Syndr. 2022 Oct 21;14:151. doi: 10.1186/s13098-022-00920-y (PMC9585795; doi:10.1186/s13098-022-00920-y)
Supplement: Supplementary file 6 — Additional file 6: Meta-analysis showing the effect of OLE supplementation on cardiovascular risk factors including studies with good quality. [file 13098_2022_920_MOESM6_ESM.docx]

| **Additional file 6: Meta-analysis showing the eﬀect of OLE supplementation on cardiovascular risk factors including studies with good quality according to the Cochrane collaboration tool** | | | | | | |
| --- | --- | --- | --- | --- | --- | --- |
|  | **Number of studies with good quality** | **Meta-analysis** | | **Heterogeneity** | | |
|  |  | **WMD (95%CI)** | **P effect** | **Q statistic** | **I^2^ (%)** | **P within group** |
| FBS | 3 | -0.90 (-2.66, 0.86) | 0.32 | 2.05 | 73.5 | 0.36 |
| TC | 6 | -4.42 (-10.68, 1.83) | 0.16 | 16.84 | 89 | 0.005 |
| TG | 6 | -9.77 (-19.00, -0.57) | 0.04 | 18.58 | 91.5 | 0.002 |
| LDL | 6 | -1.60 (-5.76, 2.55) | 0.45 | 10.33 | 81.6 | 0.07 |
| HDL | 6 | 0.71 (-1.07, 2.48) | 0.43 | 15.57 | 88.5 | 0.008 |
| SBP | 4 | -3.07 (-5.38, -0.76) | 0.009 | 3.01 | 68.1 | 0.39 |
| DBP | 4 | -0.56 (-2.85, 1.74) | 0.63 | 6.60 | 86.3 | 0.09 |
